# Comparative analysis of 7 short-read sequencing platforms using the Korean Reference Genome: MGI and Illumina sequencing benchmark for whole-genome sequencing

**DOI:** 10.1093/gigascience/giab014

**Published:** 2021-03-12

**Authors:** Hak-Min Kim, Sungwon Jeon, Oksung Chung, Je Hoon Jun, Hui-Su Kim, Asta Blazyte, Hwang-Yeol Lee, Youngseok Yu, Yun Sung Cho, Dan M Bolser, Jong Bhak

**Affiliations:** Clinomics Inc., Ulsan National Institute of Science and Technology (UNIST), UNIST-gil 50, Eonyang-eup, Ulju-gun, Ulsan, 44919, Republic of Korea; Korean Genomics Center (KOGIC), Ulsan National Institute of Science and Technology (UNIST), UNIST-gil 50, Eonyang-eup, Ulju-gun, Ulsan, 44919, Republic of Korea; Department of Biomedical Engineering, School of Life Sciences, Ulsan National Institute of Science and Technology (UNIST), UNIST-gil 50, Eonyang-eup, Ulju-gun, Ulsan, 44919, Republic of Korea; Clinomics Inc., Ulsan National Institute of Science and Technology (UNIST), UNIST-gil 50, Eonyang-eup, Ulju-gun, Ulsan, 44919, Republic of Korea; Clinomics Inc., Ulsan National Institute of Science and Technology (UNIST), UNIST-gil 50, Eonyang-eup, Ulju-gun, Ulsan, 44919, Republic of Korea; Korean Genomics Center (KOGIC), Ulsan National Institute of Science and Technology (UNIST), UNIST-gil 50, Eonyang-eup, Ulju-gun, Ulsan, 44919, Republic of Korea; Korean Genomics Center (KOGIC), Ulsan National Institute of Science and Technology (UNIST), UNIST-gil 50, Eonyang-eup, Ulju-gun, Ulsan, 44919, Republic of Korea; Department of Biomedical Engineering, School of Life Sciences, Ulsan National Institute of Science and Technology (UNIST), UNIST-gil 50, Eonyang-eup, Ulju-gun, Ulsan, 44919, Republic of Korea; Clinomics Inc., Ulsan National Institute of Science and Technology (UNIST), UNIST-gil 50, Eonyang-eup, Ulju-gun, Ulsan, 44919, Republic of Korea; Clinomics Inc., Ulsan National Institute of Science and Technology (UNIST), UNIST-gil 50, Eonyang-eup, Ulju-gun, Ulsan, 44919, Republic of Korea; Clinomics Inc., Ulsan National Institute of Science and Technology (UNIST), UNIST-gil 50, Eonyang-eup, Ulju-gun, Ulsan, 44919, Republic of Korea; Geromics Ltd., 222 Mill Road, Cambridge, CB1 3NF, United Kingdom; Clinomics Inc., Ulsan National Institute of Science and Technology (UNIST), UNIST-gil 50, Eonyang-eup, Ulju-gun, Ulsan, 44919, Republic of Korea; Korean Genomics Center (KOGIC), Ulsan National Institute of Science and Technology (UNIST), UNIST-gil 50, Eonyang-eup, Ulju-gun, Ulsan, 44919, Republic of Korea; Department of Biomedical Engineering, School of Life Sciences, Ulsan National Institute of Science and Technology (UNIST), UNIST-gil 50, Eonyang-eup, Ulju-gun, Ulsan, 44919, Republic of Korea; Geromics Ltd., 222 Mill Road, Cambridge, CB1 3NF, United Kingdom; Personal Genomics Institute (PGI), Genome Research Foundation, Osong saengmyong1ro, Cheongju, 28160, Republic of Korea

**Keywords:** DNBSEQ-T7, whole-genome sequencing, sequencing platform comparison

## Abstract

**Background:**

DNBSEQ-T7 is a new whole-genome sequencer developed by Complete Genomics and MGI using DNA nanoball and combinatorial probe anchor synthesis technologies to generate short reads at a very large scale—up to 60 human genomes per day. However, it has not been objectively and systematically compared against Illumina short-read sequencers.

**Findings:**

By using the same KOREF sample, the Korean Reference Genome, we have compared 7 sequencing platforms including BGISEQ-500, DNBSEQ-T7, HiSeq2000, HiSeq2500, HiSeq4000, HiSeqX10, and NovaSeq6000. We measured sequencing quality by comparing sequencing statistics (base quality, duplication rate, and random error rate), mapping statistics (mapping rate, depth distribution, and percent GC coverage), and variant statistics (transition/transversion ratio, dbSNP annotation rate, and concordance rate with single-nucleotide polymorphism [SNP] genotyping chip) across the 7 sequencing platforms. We found that MGI platforms showed a higher concordance rate for SNP genotyping than HiSeq2000 and HiSeq4000. The similarity matrix of variant calls confirmed that the 2 MGI platforms have the most similar characteristics to the HiSeq2500 platform.

**Conclusions:**

Overall, MGI and Illumina sequencing platforms showed comparable levels of sequencing quality, uniformity of coverage, percent GC coverage, and variant accuracy; thus we conclude that the MGI platforms can be used for a wide range of genomics research fields at a lower cost than the Illumina platforms.

## Introduction

Recently, owing to rapid technological advancement, the second- and third-generation sequencing platforms can produce a large amount of short- or long-read data at relatively low cost [1]. Depending on the application, these sequencers offer several distinct advantages. Short-read–based second-generation sequencing can be used to efficiently and accurately identify genomic variations. Long-read–based third-generation sequencing can be used to identify structural variations and build high-quality *de novo* genome assemblies [2]. Short-read sequencing technologies are routinely used in large-scale population analyses and molecular diagnostic applications because of the low cost and high accuracy [3]. The recent platforms from Illumina are the HiSeqX10 and NovaSeq6000 short-read sequencers. A competing sequencer developed by Complete Genomics and MGI Tech is the DNBSEQ-T7 (formerly known as MGISEQ-T7). DNBSEQ-T7 is a new sequencing platform following on from BGISEQ-500, which uses DNA nanoball and combinatorial probe anchor synthesis to generate short reads at a very large scale [4].

In 2017 the first article was published showing similar accuracy of single-nucleotide polymorphism (SNP) detection for the BGISEQ-500 platform compared to the HiSeq2500 [5]. While the overall quality of the data generated by BGISEQ-500 was shown to be high, some of its characteristics showed lower quality compared to Illumina HiSeq2500. In addition, the comparison results for DNA, RNA, and metagenome sequencing of the Illumina and the MGI platforms have been reported [6–8]. Furthermore, coronavirus analysis studies using an MGI platform have been reported in 2020 [9, 10]. Despite this, to date no study has compared Illumina platforms with DNBSEQ-T7 for whole-genome sequencing (WGS). In the present study, we compared 7 short-read–based sequencers: 2 MGI platforms (BGISEQ-500 and DNBSEQ-T7) and 5 Illumina platforms (HiSeq2000, HiSeq2500, HiSeq4000, HiSeqX10, and NovaSeq6000) (Table 1). We focused on how similar the 2 sets of platforms are rather than the accuracy of each sequencer, by comparing variants and platform-specific covered regions, as well as the concordance rate to SNP genotyping chip.

**Table 1:** Raw read statistics for 7 sequencing platforms

Metric	Illumina platforms					MGI platforms	
	HiSeq2000	HiSeq2500	HiSeq4000	HiSeqX10	NovaSeq6000	BGISEQ-500	DNBSEQ-T7
Production date	2012	2015.03	2015.10	2015.12	2019.04	2017.04	2019.09
Quality range	Illumina 1.5+	Illumina 1.8+	Illumina 1.8+	Illumina 1.8+	Illumina 1.8+	Illumina 1.8+	Illumina 1.8+
No. of total reads (millions)	1,044	1,500	629	833	833	1,171	1,035
PE read length (bp)	90	101	151	151	151	100	100
Total bases (Gb)	94	151.5	95	125.8	125.8	117.1	103.4
Sequencing depth (×, based on 3 Gb)	31.31	50.52	31.65	41.94	41.94	39.04	34.49

## Results

### Sequencing data summary

We analyzed and benchmarked the WGS data quality generated by the 7 sequencers using the KOREF (the Korean Reference Genome) [11] DNA. Owing to the sequential release and distribution of the sequencers, KOREF sequencing has been carried out in the 9 years following the project's launch in 2010. Therefore, the blood samples, library construction, and sequencing conditions were not the same, although all the samples were from 1 individual. The Illumina platform data used here were from 2012–2019, while the MGI platform data were from 2017 and 2019. The read length differed depending on the platform. The Illumina HiSeq2000 had the shortest read length of 90 bp paired-end (PE) and the HiSeq4000, HiSeqX10, and NovaSeq6000 had 151 bp PE. The read length of the HiSeq2500 is 101 bp PE, and that of the BGISEQ-500 and DNBSEQ-T7 is 100 bp PE. Additionally there is a difference in the amount of data produced, so we therefore randomly selected 35× coverage sequencing data for HiSeq2500 and NovaSeq6000, which have equivalent amounts of sequencing data matching that of the BGISEQ-500 and HiSeqX10 platforms. HiSeq2000, HiSeq4000, and DNBSEQ-T7 had ∼30× coverage.

### Assessment of base quality and sequencing error in raw reads

Base quality is an important factor in evaluating the performance of sequencing platforms. We analyzed the sequencing quality by identifying low-quality reads. First, we investigated the base quality distribution of raw reads with FastQC (FastQC, RRID:SCR_014583) [12]. All 7 sequencing platforms showed that the quality of each nucleotide gradually decreased towards the end of a read (Supplementary Fig. S1). The quality value of the HiSeq4000 and HiSeqX10 reads showed a tendency to decrease rapidly towards the end of the read. We defined low-quality reads as those that had >30% of bases with a sequencing quality score <20. The fraction of low-quality reads ranged from 2.8% to 18.3% across the 7 sequencing platforms (Supplementary Fig. S2 and Table S1). On the basis of the filtering criteria, the newest platforms, NovaSeq6000 and DNBSEQ-T7, showed the lowest percentage of low-quality reads (2.8% and 4.2%, respectively).

**Figure 1:**
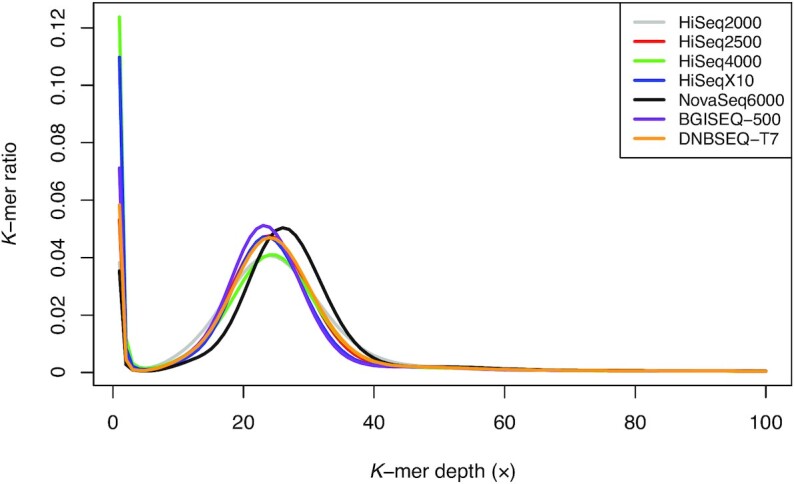
Distribution of *k*-mer frequency for 21-mers using raw reads from 7 sequencing platforms. The x-axis represents *k*-mer depth, and the y-axis represents the proportion of *k*-mers, as calculated by the frequency at that depth divided by the total frequency at all depths.

We analyzed the frequency of random sequencing errors (ambiguous base, N), which is also an important factor to evaluate the quality of the sequencing platform. We found that the HiSeq2000, HiSeq4000, and HiSeqX10 showed a high random error ratio in certain sequencing cycles (Supplementary Fig. S3 and Table S2). Furthermore, in the case of HiSeq2000, the random error tended to increase gradually after each sequencing cycle. We also investigated the sequencing error using *k*-mer analysis. Most erroneous *k*-mers caused by sequencing error appeared at very low frequency and formed a sharp left-side peak [13, 14]. The *k*-mer frequencies showed similar distributions between the platforms (Fig. 1). However, there was a difference in the proportion of low-frequency *k*-mers (≤3 *k*-mer depth), which was attributed to putative sequencing errors (Supplementary Table S3). The NovaSeq6000 showed the lowest amount of erroneous *k*-mers (3.91%), while the HiSeq4000 contained the highest amount of erroneous *k*-mers (13.91%) among the 7 sequencing platforms. The BGISEQ-500 and DNBSEQ-T7 showed a moderate level of erroneous *k*-mers (7.72% and 6.39%, respectively).

We examined the duplication rate and adapter contamination in the 7 sequencing platforms (Supplementary Table S2). We examined the exact duplicates, which are identical sequence copies, from raw sequence data. The HiSeq2000 and DNBSEQ-T7 showed the highest duplicate ratio (8.71% in HiSeq2000 and 3.04% in DNBSEQ-T7). The HiSeq4000, HiSeqX10, and NovaSeq6000 showed higher adapter contamination rates than other platforms, probably due to longer sequence length (151 bp). However, duplicates and adapter contamination may be more affected by the process of sample preparation than by the sequencing instrument.

### Genome coverage and sequencing uniformity

To assess genomic coverage and sequencing uniformity, we aligned quality-filtered reads to the human reference genome (GRCh38). All 7 sequencing platforms showed a mapping rate of >99.98% and genome coverage of >99.6% (≥1×; Table 2). We observed a higher duplicate mapping rate in the HiSeq2000 (15.35%) and DNBSEQ-T7 (8.77%) than the other platforms and the same pattern as the duplication rates of raw reads (see Supplementary Table S2). Additionally, it was also observed that duplication rates of other DNBSEQ-T7 data were also high, which were generated by the same run with the KOREF data (Supplementary Table S4). The insert size for PE libraries corresponds to the targeted fragment size for each platform (Supplementary Fig. S4). It has been reported that the depth of coverage is often far from evenly distributed across the sequenced genome [15]. To assess the sequencing uniformity, we analyzed the distribution of mapping depth for all chromosomes (Supplementary Fig. S5). All 7 platforms showed a similar pattern of depth distribution, but interestingly, we found that the depth near the centromere regions was lower exclusively in the HiSeq4000 (Supplementary Figs. S6–S9). We speculate that this may have been due to a bias in the library preparation step on the HiSeq4000 platform.

**Table 2:** Mapping and coverage statistics

Metric	HiSeq2000	HiSeq2500	HiSeq4000	HiSeqX10	NovaSeq6000	BGISEQ-500	DNBSEQ-T7
No. of clean reads	935,951,974	1,050,028,628	512,891,970	705,987,420	706,000,000	1,060,837,856	991,021,996
Read length	90	101	151	151	151	100	100
Clean bases (Gb)	84.23	106.05	77.45	106.60	106.6	106.08	99.1
Clean read depth (based on 3 Gb, ×)	28.08	35.35	25.82	35.53	35.54	35.36	33.03
Mapping rate (%)	99.986	99.999	99.990	99.999	99.9996	99.983	99.999
Properly mapped rate (%)*	96.67	98.30	97.24	96.91	97.15	97.44	98.17
Duplicate rate (%)	15.35	3.01	3.19	5.08	3.39	2.56	8.77
Duplicate clean read depth (×)	23.90	34.29	24.99	33.73	34.33	34.46	30.14
Down-sampled depth (×)	23.90	23.90	23.90	23.90	23.90	23.90	23.90
Coverage (%)							
Any	99.68	99.82	99.71	99.81	99.76	99.83	99.83
≥5×	98.62	99.30	98.37	99.30	99.19	99.34	99.24
≥10×	94.63	96.65	93.98	97.05	96.89	97.05	96.61
≥15×	85.10	88.54	85.08	90.23	90.36	90.11	89.36

* Both of the read mates are in the correct orientation.

To examine the platform-specific covered region of the MGI and Illumina platforms, we defined a platform-specific covered region that had significantly different depths based on the 100-bp non-overlapping windows and statistical test [16]. Prior to examining the platform-specific covered regions, mapped reads were down-sampled for all platforms to 24× coverage, which is the minimum coverage among the platforms, for a fair comparison (Supplementary Table S5). We found 178 and 297 kb of the platform-specific covered regions from the MGI and Illumina platforms, respectively (Supplementary Table S6). A total of 168 and 373 genes were overlapped in MGI- and Illumina-specific covered regions, respectively, and most of them were intronic. Interestingly, however, the platform-specific covered regions showed a significantly different distribution of GC ratios between the MGI and Illumina platforms (Supplementary Fig. S10). The MGI platforms tend to cover regions relatively high in GC content (Wilcoxon rank-sum test, *P* = 2.37 × 10^−133^). Nevertheless, it is obvious that platform-specific covered regions for Illumina platforms are slightly longer than those of the MGI platforms, and these regions were not sufficiently covered by the MGI platforms.

**Figure 2:**
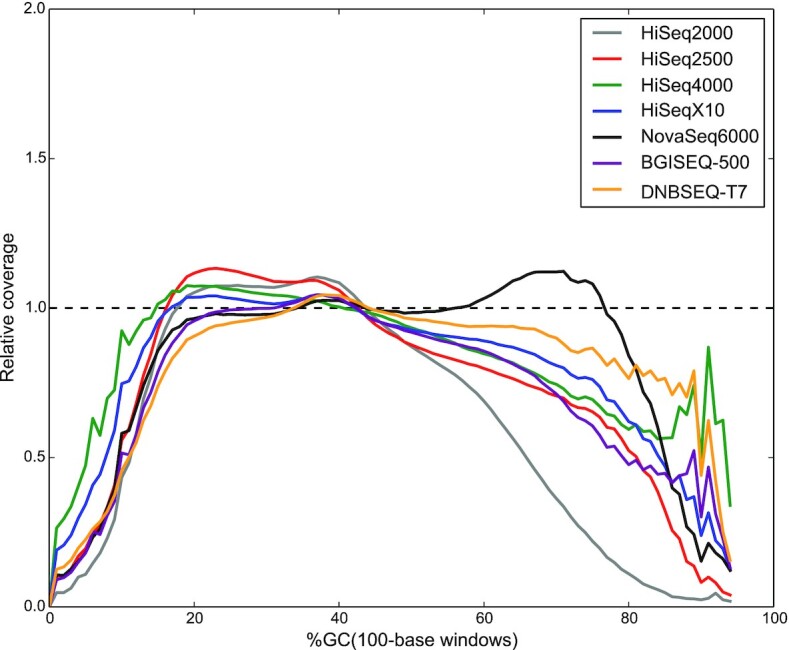
GC-bias plots for 7 sequencing platforms. Unbiased coverage is represented by a horizontal dashed line at relative coverage = 1. A relative coverage <1 indicates lower than expected coverage and >1 indicates higher than expected coverage.

Biases in PCR amplification create uneven genomic representation in classic Illumina libraries [17, 18] because PCR is sensitive to extreme GC-content variation [19]. Thus, we analyzed the GC biases for 7 sequencing platforms. We examined the distribution of GC content in sequencing reads and found that raw reads of all 7 sequencing platforms showed a similar GC content distribution to the human reference genome (Supplementary Fig. S11). To better understand what parts of the genome were not covered properly, we generated GC-bias plots, showing relative coverage at each GC level. Unbiased sequencing would not be affected by GC composition, resulting in a flat line along with relative coverage = 1. We found that all 7 sequencing platforms provided nearly even coverage in the moderate-GC range 20–60%, which represents ∼95% of the human genome (Fig. 2). On the other hand, the relative coverage of the HiSeq2000 platform decreased faster above 60% GC than other platforms, while the NovaSeq6000 covered well above 60% GC, unlike the other platforms.

### Comparison of variants detected among 7 sequencing platforms

**Figure 3:**
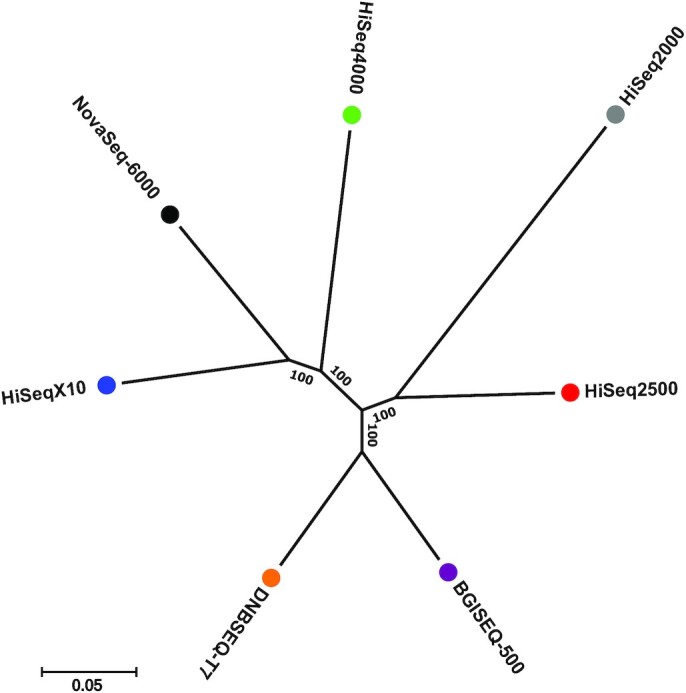
An unrooted tree for 7 sequencing platforms showing the similarity of the variant calling. Numbers of nodes denote bootstrap values based on 1,000 replicates.

To investigate the performance of variant calling for the 7 sequencing sequencers, we adopted the widely used pipeline BWA-MEM (BWA, RRID:SCR_010910) [20] and GATK (GATK, RRID:SCR_001876) [21–23]. We identified an average of 4.14 million single-nucleotide variants (SNVs) and 0.61 million indels (insertion and deletion) on each of the 7 sequencing platforms (Table 3). The statistics of SNVs were similar across all 7 in terms of the dbSNP annotation rate (dbSNP153) and the transition/transversion (Ti/Tv) ratio, which indirectly reflects SNV-calling accuracy. Approximately 3.7 million SNV loci were found on all 7 sequencing platforms, and this accounts for 87–91% of the discovered SNVs on each platform (Supplementary Table S7). We found 13,999 and 9,691 platform-specific SNVs on the MGI and Illumina platforms, respectively. To figure out the potential cause of the platform-specific SNVs, we checked how many of the SNVs were located on the platform-specifically covered regions. Only 2.8% of Illumina platform-specific SNVs and 1.6% of MGI platform-specific SNVs were located on the platform-specifically covered region (Supplementary Table S8), and most of the platform-specific SNVs were located on regions with sufficient sequencing depths (>10×). It was also found that ∼74% of platform-specific SNVs were located on the repeat region (Supplementary Table S9). The number of singletons, variations found only in 1 platform, was higher for the Illumina (∼0.10 million SNVs on average) than MGI (∼0.05 million SNVs on average) sequencers (see Supplementary Table S7). This means that the difference within the Illumina platforms is greater than the difference between the MGI platforms. Similar to the case of the platform-specific SNVs, a few singletons were found in the platform-specific covered region (0.5% on average), and most of the singletons were located on sufficiently high sequencing depth regions (>10×, Supplementary Table S10). Approximately 74% of singletons were located on the repeat region (see Supplementary Table S9). We speculate that the repeat region is one of the sources causing the platform-specific SNVs and singletons. We also analyzed the number of SNVs found in any 6 of the 7 sequencing platforms, which we considered fase-negative calls (Supplementary Table S11). The HiSeq2000 had the largest number of false-negative calls (64,856 SNVs) among the 7 sequencing platforms. The 2 MGI platforms (DNBSEQ-T7 and BGISEQ-500) had 18,826 and 15,657 false-negative calls, respectively, and those of the NovaSeq6000 showed the smallest number of false-negative calls (6,999 SNVs). To investigate the relationship between the sequencing platforms, an unrooted tree was constructed using a total of 1,036,417 loci where the genotypes of 1 or more platforms differ from the rest of the platforms (Fig. 3 and Supplementary Table S12). We found that the 2 MGI platforms grouped together, and they are the closest to the Illumina HiSeq2500 platform. The Illumina platforms were divided into 2 subgroups in the tree: a long read length (151 bp) group containing the HiSeq4000, HiSeqX10, and NovaSeq6000 platforms and a short read length (≤101 bp) group containing the HiSeq2000 and HiSeq2500 platforms. Read length primarily affects the detection of variants through alignment bias and alignment errors, which are higher for short reads because there is less chance of a unique alignment to the reference sequence than with longer reads [24].

**Table 3:** Variant statistics of Illumina and MGI sequencing platforms

Metric	HiSeq2000	HiSeq2500	HiSeq4000	HiSeqX10	NovaSeq6000	BGISEQ-500	DNBSEQ-T7
Reference homozygous	2,839,358,003	2,855,619,759	2,855,062,233	2,864,272,103	2,861,198,782	2,851,898,568	2,853,066,635
No. of no call positions	80,241,142	63,980,549	64,532,078	55,244,498	58,311,103	67,747,107	66,584,361
No call rate (%)	2.74	2.19	2.21	1.89	1.99	2.32	2.28
SNVs							
Total	4,133,925	4,132,468	4,138,296	4,216,589	4,223,612	4,088,645	4,082,103
Total in dbSNP	4,094,212	4,114,993	4,112,253	4,198,005	4,184,100	4,070,101	4,064,986
dbSNP rate (%)	99.04	99.58	99.37	99.56	99.06	99.55	99.58
Singletons	159,429	78,109	98,574	100,158	104,052	52,127	51,978
Singletons in dbSNP	126,762	68,673	78,361	89,094	73,177	41,092	41,743
dbSNP rate for singletons (%)	79.51	87.92	79.49	88.95	70.33	78.83	80.31
Homozygous	1,703,616	1,690,878	1,704,813	1,708,639	1,714,752	1,688,328	1,689,834
Heterozygous	2,430,309	2,441,590	2,433,483	2,507,950	2,508,860	2,400,317	2,392,269
Heterozygous/homozygous ratio	1.43	1.44	1.43	1.47	1.46	1.42	1.42
Ti/Tv ratio	1.91	1.92	1.9	1.88	1.85	1.92	1.92
Indels							
Total	526,504	546,918	491,899	689,357	708,062	703,873	631,163
Total in dbSNP	524,738	544,866	489,777	686,916	705,553	701,802	629,314
dbSNP rate (%)	99.66	99.62	99.57	99.65	99.65	99.71	99.71
Singletons	7,864	7,444	8,094	17,036	23,596	41,384	12,092
Singletons in dbSNP	7,612	7,259	7,915	16,784	23,303	41,183	11,964
dbSNP rate for singletons (%)	96.80	97.51	97.79	98.52	98.76	99.51	98.94

Because it was not possible to conduct standard benchmarking procedures and determine error values for each platform in this study, we compared the variations called by the 7 whole-genome sequences with an SNP genotyping chip as an independent platform. Of the total 950,585 comparable positions, >99.3% of the genotypes matched the WGS-based genotypes from the 7 sequencing platforms (Supplementary Table S13). We found that 4,356 loci in the SNP genotyping were inconsistent across all 7 WGS-based genotyping results, suggesting that these loci are probably errors in the SNP genotyping chip. With the exception of HiSeq2000 and HiSeq4000, all the other platforms showed a similar concordance rate.

## Discussion

Our benchmarks can provide a useful but rough estimation of the quality of short-read–based whole-genome sequencers. We used the same individual's samples for all 7 sequencing platforms, but these were collected at different time points over the past 7 years. Just 1 human sample cannot justify the variation that may occur among different individuals, extracted DNA molecules, and overall sequencing qualities. Furthermore, the sequencing quality may vary greatly depending on the version of the library preparation kit, even on the same platform [25]. These are clear limitations of our benchmarking; however, because our purpose was to compare 2 major platforms, namely, Illumina and MGI, the whole-genome data from just 1 individual can function as an intuitive index for researchers who are considering purchasing large sequencers to generate a very large amount of sequencing data (Supplementary Table S14). Our method of statistical analysis does not allow us to conclude which of the 7 sequencing instruments is the most accurate and precise because there is much variation in the sample preparation and sequencer specifications. Nevertheless, overall, the data generated by the Illumina and MGI sequencing platforms showed comparable levels of quality, sequencing uniformity, percent GC coverage, and concordance rate with SNP genotyping; thus it can be broadly concluded that the MGI platforms can be used for a wide range of research tasks on a par with Illumina platforms, and at a lower cost [7].

## Materials and Methods

### Genomic DNA extraction and SNP genotyping

Genomic DNA used for genotyping and sequencing were extracted from the peripheral blood of a Korean male sample donor (KOREF). The genomic DNA was extracted using the DNeasy Blood & Tissue kit (Qiagen, Valencia, CA, USA) according to the manufacturer's recommendations. DNA quality was assessed by running 1 μL on the Bioanalyzer system (Agilent, Santa Clara, CA, USA) to ensure size and analysis of DNA fragments. The concentration of DNA was assessed using the dsDNA BR assay on a Qubit fluorometer (Thermo Fisher Scientific, Carlsbad, CA, USA). We conducted a genotyping experiment using the Illumina Infinium Omni1 quad chip according to the manufacturer's protocols. The Institutional Review Board (IRB) at Ulsan National Institute of Science and Technology approved the study (UNISTIRB-15–19-A).

### Illumina PE library construction and sequencing

High molecular weight genomic DNA was sheared using a Covaris S2 ultra sonicator system to get appropriate sizes (Covaris, Woburn, MA, USA). Libraries with short inserts of 500 bp for HiSeq2000, 400 bp for HiSeq2500 (HiSeq2500, RRID:SCR_016383) and HiSeq4000 (HiSeq4000, RRID:SCR_016386), and 450 bp for HiSeqX10 and NovaSeq6000 for PE reads were prepared using TruSeq DNA sample prep kit following the manufacturer's protocol. Products were quantified using the Bioanalyzer (Agilent, Santa Clara, CA, USA) and the raw data were generated by each Illumina platform. Further image analysis and base calling were conducted with the Illumina pipeline using default settings.

### MGI PE library construction and sequencing

The KOREF genomic DNA was fragmented by Frag enzyme (MGI, Shenzhen, China) to DNA fragments between 100 and ∼1,000 bp suitable for PE100 sequencing according to the manufacturer's instructions (MGI FS DNA library prep set, cat No. 1,000,005,256). The fragmented DNA was further selected to be between 300 and ∼500 bp by DNA clean beads (MGI, Shenzhen, China). The selected DNA fragments were then repaired to obtain a blunt end and modified at the 3′ end to get a dATP as a sticky end. The dTTP tailed adapter sequence was ligated to both ends of the DNA fragments. The ligation product was then amplified for 7 cycles and subjected to the following single-strand circularization process. The PCR product was heat-denatured together with a special molecule that was reverse-complemented to 1 special strand of the PCR product, and the single-strand molecule was ligated using DNA ligase. The remaining linear molecule was digested with the exonuclease, finally obtaining a single-strand circular DNA library. We sequenced the DNA library using BGISEQ-500 (BGISEQ-500, RRID:SCR_017979) and DNBSEQ-T7 (DNBSEQ-T7, RRID:SCR_017981) with a PE read length of 100 bp.

### Raw data preprocessing

We used FastQC v0.11.8 (FastQC, RRID:SCR_014583) [12] to assess overall sequencing quality for MGI and Illumina sequencing platforms. PCR duplications (reads were considered duplicates when forward read and reverse read of the 2 PE reads were identical) were detected by PRINSEQ v0.20.4 (PRINSEQ, RRID:SCR_005454) [26]. The random sequencing error rate was calculated by measuring the occurrence of “N” bases at each read position in raw reads. Reads with sequencing adapter contamination were examined according to the manufacturer's adapter sequences (Illumina sequencing adapter left = “GATCGGAAGAGCACACGTCTGAACTCCAGTCAC,” Illumina sequencing adapter right = “GATCGGAAGAGCGTCGTGTAGGGAAAGAGTGT,” MGI sequencing adapter left = “AAGTCGGAGGCCAAGCGGTCTTAGGAAGACAA,” and MGI sequencing adapter right = “AAGTCGGATCGTAGCCATGTCGTTCTGTGAGCCAAGGAGTTG”). We conducted base quality filtration of raw reads using the NGS QC Toolkit v2.3.3 (cut-off read length for high quality 70; cut-off quality score, 20) (NGS QC Toolkit, RRID:SCR_005461) [27]. We used clean reads after removing low-quality reads and adapter-containing reads for the mapping step.

### Mapping, variant calling, and coverage calculation

After the filtering step, clean reads were aligned to the human reference genome (GRCh38) using BWA-MEM v0.7.12, and duplicate reads were removed using Picard v2.6.0 (Picard, RRID:SCR_006525) [28]. After removing duplicate reads, we down-sampled the deduplicated clean reads of all the sequencing platforms to 24× coverage according to the amount of the deduplicated clean reads of HiSeq2000 for a fair comparison. Realignment and base score recalibration of the bam file was processed by GATK v3.3. SNVs, short insertions, and deletions were called with GATK (Unifiedgenotyper, options –output_mode EMIT_ALL_SITES –genotype_likelihoods_model BOTH). The resulting variants were annotated with the dbSNP (v153) database [29]. Coverage was calculated for each nucleotide using SAMtools v1.9 (SAMTOOLS, RRID:SCR_002105) [30]. We defined a specific covered region based on the 100-bp non-overlapping windows by calculating the average depth of the windows followed by a statistical test. We used the edgeR method as the statistical test [16]. *P*-values were adjusted by Benjamini-Hochberg correction. GC coverage for raw reads and the genome was calculated by the average percent GC of the 100-bp non-overlapping windows.

### Variant comparison and concordance rate with SNP genotyping

The chromosome position and genotype of each variant called from each sequencing platform was used to identify the relationship between the 7 sequencing platforms. We compared 1,036,417 loci found on 1 or more platforms for locations where genotypes were determined on all 7 platforms. An unrooted tree was generated using FastTree v2.1.10 (FastTree, RRID:SCR_015501) [31] with the generalized time-reversible (GTR) model. To calculate the concordance rate between SNP genotyping and WGS-based genotype, the coordinates of the SNP genotyping data were converted to GRCh38 assembly using the UCSC LiftOver tool [32]. We removed unmapped positions and indel markers and used only markers that were present on the autosomal chromosomes.

## Data Availability

All sequences generated in this study, including the HiSeq2000, HiSeq2500, HiSeq4000, HiSeqX10, NovaSeq6000, BGISEQ-500, and DNBSEQ-T7 sequencing reads, were deposited in the NCBI SRA database under BioProject PRJNA600063. All benchmarking data are hosted and distributed from the BioSequencer home page [33], and supporting data and materials are also available at *GigaScience* GigaDB [34].

## Additional Files

**Supplementary Figure S1**. Distribution of nucleotide quality across 7 sequencing platforms.

**Supplementary Figure S2**. Base quality filtration statistics for 7 sequencing platforms.

**Supplementary Figure S3**. Random error ratio for 7 sequencing platforms.

**Supplementary Figure S4**. Insert size distributions for 7 sequencing platforms.

**Supplementary Figure S5**. The coverage distribution of 2 MGI and 5 Illumina platforms.

**Supplementary Figure S6**. Depth distribution of chromosome 8.

**Supplementary Figure S7**. Depth distribution of chromosome 12.

**Supplementary Figure S8**. Depth distribution of chromosome 18.

**Supplementary Figure S9**. Depth distribution of chromosome 20.

**Supplementary Figure S10**. GC distribution of platform-specific covered regions.

**Supplementary Figure S11**. The GC composition distribution of the human genome and sequencing reads.

**Supplementary Table S1**. Base quality summary.

**Supplementary Table S2**. Duplicate reads, random error base, and adapter read rate.

**Supplementary Table S3**. The putatively erroneous *k*-mers (≤3 *k*-mer depth) for 7 sequencing platforms.

**SupplementaryTable S4**. Mapping and duplicate rate of samples using MGI's PE100 protocol and DNBSEQ-T7.

**SupplementaryTable S5**. Statistics of clean reads for 7 sequencing platforms.

**Supplementary Table S6**. Statistics for platform-specific covered regions.

**Supplementary Table S7**. The number of shared SNVs for 7 sequencing platforms.

**Supplementary Table S8**. Statistics of platform-specific SNVs.

**Supplementary Table S9**. Statistics of platform-specific SNVs and singletons in the repeat region.

**Supplementary Table S10**. Statistics of singleton variants.

**Supplementary Table S11**. The number of SNVs not found on a specific platform.

**Supplementary Table S12**. Genotype concordance rate among 7 sequencing platforms.

**Supplementary Table S13**. Genotype comparison between SNP genotyping and WGS.

**Supplementary Table S14**. Recent studies for MGI and Illumina platform comparison.

## Abbreviations

bp: base pairs; BWA: Burrows-Wheeler Aligner; GATK: Genome Analysis Toolkit; Gb: gigabase pairs; GC: guanine-cytosine; indels: insertions and deletions; kb: kilobase pairs; KOREF: Korean Reference Genome; PE: paired-end; SNP: single-nucleotide polymorphism; SNV: single-nucleotide variant; SRA: Sequence Read Archive; Ti/Tv: transition/transversion; WGS: whole-genome sequencing.

## Competing Interests

H.M.K., O.C., Y.S.C., J.H.J., H.Y.L., and Y.Y. are employees and J.B. is the chief executive officer of Clinomics Inc. H.M.K., Y.S.C., and J.B. have an equity interest in the company. All other authors declare that they have no competing interests.

## Funding

This work was supported by the Research Project Funded by the Ulsan City Research Fund (1.200047.01) of the Ulsan National Institute of Science & Technology (UNIST) and Clinomics and Geromics Ltd internal funding. This work was also supported by the Technology Innovation Program (20010587, Development and Dissemination on National Standard Reference Data) funded by the Ministry of Trade, Industry & Energy (MOTIE, Korea).

## Authors’ Contributions

J.B. supervised and coordinated the project. J.B. and Y.S.C. conceived and designed the experiments. H.M.K., S.J., O.C., J.H.J., H.Y.L., and Y.Y. conducted the bioinformatics data processing and analyses. H.M.K., S.J., D.M.B., and J.B. wrote and revised the manuscript. A.B. and H.S.K. reviewed and edited the manuscript. All authors have read and approved the final manuscript.

## Supplementary Material

giab014_GIGA-D-20-00072_Original_Submission

giab014_GIGA-D-20-00072_Revision_1

giab014_GIGA-D-20-00072_Revision_2

giab014_Response_to_Reviewer_Comments_Original_Submission

giab014_Response_to_Reviewer_Comments_Revision_1

giab014_Reviewer_1_Report_Original_Submission Dan Xie -- 5/11/2020 Reviewed

giab014_Reviewer_1_Report_Revision_1 Dan Xie -- 9/24/2020 Reviewed

giab014_Reviewer_2_Report_Original_Submission Xuming Zhou -- 5/29/2020 Reviewed

giab014_Reviewer_2_Report_Revision_1 Xuming Zhou -- 9/21/2020 Reviewed

giab014_Reviewer_2_Report_Revision_2 Xuming Zhou -- 1/14/2021 Reviewed

giab014_Reviewer_3_Report_Original_Submission Inge Seim -- 6/4/2020 Reviewed

giab014_Supplemental_File
