# Supplementary material for: Comparative analysis of 7 short-read sequencing platforms using the Korean Reference Genome: MGI and Illumina sequencing benchmark for whole-genome sequencing
Source: Gigascience. 2021 Mar 12;10(3):giab014. doi: 10.1093/gigascience/giab014 (PMC7953489; doi:10.1093/gigascience/giab014)
Supplement: giab014_GIGA-D-20-00072_Revision_2 [file giab014_giga-d-20-00072_revision_2.pdf]

GigaScience

Comparative analysis of seven short-read sequencing platforms using the Korean Reference Genome: MGI and Illumina sequencing benchmark for whole-genome sequencing

--Manuscript Draft--

Manuscript Number:	GIGA-D-20-00072R2
Full Title:	Comparative analysis of seven short-read sequencing platforms using the Korean Reference Genome: MGI and Illumina sequencing benchmark for whole-genome sequencing
Article Type:	Data Note
Funding Information:	Ulsan National Institute of Science and Technology (1.200047.01) — Dr. Jong Hwa Bhak<br>Ministry of Trade, Industry and Energy (20010587) — Dr. Jong Hwa Bhak
Abstract:	Background: DNBSEQ-T7 is a new whole-genome sequencer developed by Complete Genomics and MGI utilizing DNA nanoball and combinatorial probe anchor synthesis technologies to generate short reads at a very large scale – up to 60 human genomes per day. However, it has not been objectively and systematically compared against Illumina short-read sequencers.<br>Findings: By using the same KOREF sample, the Korean Reference Genome, we have compared seven sequencing platforms including BGISEQ-500, DNBSEQ-T7, HiSeq2000, HiSeq2500, HiSeq4000, HiSeqX10, and NovaSeq6000. We measured sequencing quality by comparing sequencing statistics (base quality, duplication rate, and random error rate), mapping statistics (mapping rate, depth distribution, and %GC coverage), and variant statistics (transition/transversion ratio, dbSNP annotation rate, and concordance rate with SNP genotyping chip) across the seven sequencing platforms. We found that MGI platforms showed a higher concordance rate for SNP genotyping than HiSeq2000 and HiSeq4000. The similarity matrix of variant calls confirmed that the two MGI platforms have the most similar characteristics to the HiSeq2500 platform.<br>Conclusions: Overall, MGI and Illumina sequencing platforms showed comparable levels of sequencing quality, uniformity of coverage, %GC coverage, and variant accuracy, thus we conclude that the MGI platforms can be used for a wide range of genomics research fields at a lower cost than the Illumina platforms.
Corresponding Author:	Jong Hwa Bhak, Ph.D.<br>UNIST<br>Ulsan, Ulsan KOREA, REPUBLIC OF
Corresponding Author Secondary Information:	
Corresponding Author's Institution:	UNIST
Corresponding Author's Secondary Institution:	
First Author:	Hak-Min Kim, Ph.D.
First Author Secondary Information:	
Order of Authors:	Hak-Min Kim, Ph.D.
	Sungwon Jeon
	Oksung Chung
	Je Hoon Jun
	Hui-Su Kim, Ph.D.
	Asta Blazyte

Powered by Editorial Manager® and ProduXion Manager® from Aries Systems Corporation

	Hwang-Yeol Lee
	Youngseok Yu
	Yun Sung Cho, Ph.D.
	Dan M. Bolser, Ph.D.
	Jong Hwa Bhak, Ph.D.
**Order of Authors Secondary Information:**	
**Response to Reviewers:**	Reviewer reports:<br><br>Reviewer #1: Much improved manuscript. I only have minor comments:<br>1) The examination of platform-specific covered region between MGI and Illumina platforms is still problematic. A single fold change threshold is unreliable. The authors should further make statistical test to identify platform-specific covered regions.<br>==> As pointed out by the reviewer, we re-analyzed the platform-specific covered region between MGI and Illumina platforms. We now use statistical test (edgeR method for group comparison followed by Benjamini-Hochberg correction for p-value adjustment) rather than the single fold change threshold to identify the platform-specifically covered region. As a result, the number of platform-specific covered regions of MGI platform increased from 1,436 to 1,778, and in the case of Illumina, increased from 2,881 to 2,967. We updated the manuscript and supplementary figure and table (See Results section lines 143-145; Figure S10 and Table S6).<br><br>2) Since the standard variant data set is not available, I think it is necessary to discuss the potential reason of the platform-specific SNVs and the singletons. Whether their distribution is associated with platform-specific covered regions or other reasons associated with low sequencing quality?<br>==> We speculate that repetitive regions with low mapping tendency were the one of the reasons for the platform-specific SNVs and singletons.<br>To figure out the potential reason of the platform-specific SNVs and the singletons, we compared these SNVs to platform-specific covered regions. First, we compared platform-specific SNVs to platform-specific covered regions. We found only 2.8% of Illumina platform-specific SNVs and 1.6% of MGI platform-specific SNVs are included in the platform specific covered region (Table S8). In addition, most of the platform-specific SNVs were located in a sufficient depth region (>10×), and about 74% of platform-specific SNVs were included in the repeat region (Table S9).<br>The singleton also showed a similar pattern to platform-specific SNVs. There were very few overlapping positions between the singleton variants and the platform-specific covered region (0.5% on average, Table S10), and most of the singletons were located in the relatively high depth region (>10×). About 74% of singletons were included in the repeat region (Table S9).<br>We updated these results to the manuscript (See Results section lines 179-194).<br><br>Reviewer #2: The authors addressed my and other reviewers's comments however many of the changes were quite minimal. It is suggested they can put the additional test in the main text and clarify all those limitations (not simple mentioned) in their study in the discussion section. For example, the high duplicate ratio in MGISEQ-T7 and a single individual was used.<br>==> Thanks for the comment. We now added additional result for platform-specific SNVs, singleton, and high duplicate ratio of MGISEQ-T7 platform in the manuscript (See Results section lines 134-135; Tables S4, S8, S9, and S10). Furthermore, we added a list of sequencing platform comparison studies using single individual in the discussion section (See Discussion section lines 221-228; Table S14).
**Additional Information:**	
**Question**	**Response**
Are you submitting this manuscript to a special series or article collection?	No

Powered by Editorial Manager® and ProduXion Manager® from Aries Systems Corporation

**Experimental design and statistics**<br><br>Full details of the experimental design and statistical methods used should be given in the Methods section, as detailed in our Minimum Standards Reporting Checklist. Information essential to interpreting the data presented should be made available in the figure legends.<br><br>Have you included all the information requested in your manuscript?	Yes
**Resources**<br><br>A description of all resources used, including antibodies, cell lines, animals and software tools, with enough information to allow them to be uniquely identified, should be included in the Methods section. Authors are strongly encouraged to cite Research Resource Identifiers (RRIDs) for antibodies, model organisms and tools, where possible.<br><br>Have you included the information requested as detailed in our Minimum Standards Reporting Checklist?	Yes
**Availability of data and materials**<br><br>All datasets and code on which the conclusions of the paper rely must be either included in your submission or deposited in publicly available repositories (where available and ethically appropriate), referencing such data using a unique identifier in the references and in the "Availability of Data and Materials" section of your manuscript.<br><br>Have you have met the above requirement as detailed in our Minimum Standards Reporting Checklist?	Yes

Powered by Editorial Manager® and ProduXion Manager® from Aries Systems Corporation

Manuscript

Click here to access/download;Manuscript;072-Sequencing_Platform_final-HL-JB.docx

Comparative analysis of seven short-read sequencing platforms using the Korean Reference Genome: MGI and Illumina sequencing benchmark for whole-genome sequencing

Hak-Min Kim[1], Sungwon Jeon[2,3], Oksung Chung[1], Je Hoon Jun[1], Hui-Su Kim[2], Asta Blazyte[2,3], Hwang-Yeol Lee[1], Youngseok Yu[1], Yun Sung Cho[1], Dan M. Bolser[4]*, and Jong Bhak[1,2,3,4,5]*

[1]Clinomics, Ulsan National Institute of Science and Technology, Ulsan 44919, Republic of Korea

[2]Korean Genomics Center (KOGIC), Ulsan National Institute of Science and Technology, Ulsan 44919, Republic of Korea

[3]Department of Biomedical Engineering, School of Life Sciences, Ulsan National Institute of Science and Technology, Ulsan 44919, Republic of Korea

[4]Geromics Ltd, 23 King Street, Cambridge, CB1 1AH, UK

[5]Personal Genomics Institute (PGI), Genome Research Foundation, Osong 28160, Republic of Korea

*Corresponding authors

Dan M. Bolser, dan@geromics.co.uk; Jong Bhak, jongbhak@genomics.org, +82-52-217-5329

Email address:

H.M.K.: howmany2@gmail.com

S.J.: jsw0061@gmail.com
O.C.: okokookk219@gmail.com
J.H.J.: junjh0701@gmail.com
H.S.K.: hskim3824@gmail.com
A.B.: blazyte.asta@gmail.com
H.Y.L.: hyeol911@gmail.com
Y.Y.: yung7449@gmail.com
Y.S.C.: joys0406@gmail.com
D.M.B.: dan@geromics.co.uk
J.B.: jongbhak@genomics.org

ORCIDs:

Hak-Min Kim[0000-0001-6066-2469]; Sungwon Jeon[0000-0002-2729-9087]; Oksung Chung[0000-0001-5003-4071]; Je Hoon Jun[0000-0002-6558-1091]; Hui-Su Kim[0000-0003-2277-638X]; Asta Blazyte[0000-0001-7309-1482]; Hwang-Yeol Lee[0000-0002-0981-1892]; Youngseok Yu[0000-0002-7313-9519]; Yun Sung Cho[0000-0003-4490-8769]; Dan M. Bolser[0000-0002-3991-0859]; Jong Bhak[0000-0002-4228-1299]

Abstract

Background: DNBSEQ-T7 is a new whole-genome sequencer developed by Complete Genomics and MGI utilizing DNA nanoball and combinatorial probe anchor synthesis

technologies to generate short reads at a very large scale – up to 60 human genomes per day. However, it has not been objectively and systematically compared against Illumina short-read sequencers. **Findings:** By using the same KOREF sample, the Korean Reference Genome, we have compared seven sequencing platforms including BGISEQ-500, DNBSEQ-T7, HiSeq2000, HiSeq2500, HiSeq4000, HiSeqX10, and NovaSeq6000. We measured sequencing quality by comparing sequencing statistics (base quality, duplication rate, and random error rate), mapping statistics (mapping rate, depth distribution, and %GC coverage), and variant statistics (transition/transversion ratio, dbSNP annotation rate, and concordance rate with SNP genotyping chip) across the seven sequencing platforms. We found that MGI platforms showed a higher concordance rate for SNP genotyping than HiSeq2000 and HiSeq4000. The similarity matrix of variant calls confirmed that the two MGI platforms have the most similar characteristics to the HiSeq2500 platform. **Conclusions:** Overall, MGI and Illumina sequencing platforms showed comparable levels of sequencing quality, uniformity of coverage, %GC coverage, and variant accuracy, thus we conclude that the MGI platforms can be used for a wide range of genomics research fields at a lower cost than the Illumina platforms.

Keywords: DNBSEQ-T7; whole-genome sequencing; sequencing platform comparison;

Introduction

Recently, due to the rapid technological advancement, the second- and third-generation sequencing platforms can produce a large amount of short- or long-read data at relatively low cost [1]. Depending on the application, these sequencers offer several distinct advantages. Short-read based second-generation sequencing can be used to efficiently and accurately identify genomic variations. Long-read based third-generation sequencing can be used to

identify structural variations and build high quality *de novo* genome assemblies [2]. Short-read sequencing technologies are routinely used in large-scale population analyses and molecular diagnostic applications because of the low cost and high accuracy [3]. The recent platforms from Illumina are the HiSeqX10 and NovaSeq6000 short-read sequencers. A competing sequencer developed by Complete Genomics and MGI Tech is the DNBSEQ-T7 (formerly known as MGISEQ-T7). DNBSEQ-T7 is a new sequencing platform following on from BGISEQ-500, that uses DNA nanoball and combinatorial probe anchor synthesis to generate short reads at a very large scale [4].

In 2017 the first paper was published showing similar accuracy of SNP detection for the BGISEQ-500 platform compared to the HiSeq2500 [5]. While the overall quality of the data generated by BGISEQ-500 was shown to be of high quality, some of its characteristics showed lower quality compared to Illumina HiSeq2500. In addition, the comparison results for DNA, RNA, and metagenome sequencing of the Illumina and the MGI platforms have been reported [6-8]. Furthermore, coronavirus analysis studies using an MGI platform have been reported in 2020 [9, 10]. Despite this, to date no study has compared Illumina platforms with DNBSEQ-T7 for whole-genome sequencing (WGS). In the present study, we compared seven short-read based sequencers; two MGI platforms (BGISEQ-500 and DNBSEQ-T7) and five Illumina platforms (HiSeq2000, HiSeq2500, HiSeq4000, HiSeqX10, and NovaSeq6000) (Table 1). We focused on how similar the two sets of platforms are rather than the accuracy of each sequencer, by comparing variants, platform-specific covered regions as well as the concordance rate to SNP genotyping chip.

Results

Sequencing data summary

We analyzed and benchmarked the whole-genome sequencing data quality generated by the seven sequencers using the KOREF (the Korean Reference Genome) [11] DNA. Due to the sequential release and distribution of the sequencers, KOREF sequencing has been carried out in the nine years following the projects launch in 2010. Therefore, the blood samples, library construction, and sequencing conditions were not the same, although all the samples were from one individual. The Illumina platform data used here were from 2012 to 2019, while the MGI platform data were from 2017 and 2019. With the read length differing depending on the platform. The Illumina HiSeq2000 had the shortest read length of 90 bp paired-end (PE) and the HiSeq4000, HiSeqX10, and NovaSeq6000 had 151 bp PE. The read length of the HiSeq2500 is 101 bp PE and that of the BGISEQ-500 and DNBSEQ-T7 is 100 bp PE. Additionally there is a difference in the amount of data produced, so we therefore randomly selected 35× coverage sequencing data for HiSeq2500 and NovaSeq6000 which have equivalent amounts of sequencing data matching that of BGISEQ-500 and HiSeqX10 platforms. HiSeq2000, HiSeq4000, and DNBSEQ-T7 had roughly 30× coverage.

Assessment of base quality and sequencing error in raw reads

Base quality is an important factor in evaluating the performance of sequencing platforms. We analyzed the sequencing quality by identifying low-quality reads. First, we investigated the base quality distribution of raw reads with the FastQC (FastQC, RRID:SCR_014583) [12]. All seven sequencing platforms showed that the quality of each nucleotide gradually decreased towards the end of a read (Fig. S1). The quality value of the HiSeq4000 and HiSeqX10 reads showed a tendency to decrease rapidly towards the end of the read. We defined low-quality reads as those that had more than 30% of bases with a sequencing quality score lower than 20. The fraction of low-quality reads ranged from 2.8% to 18.3% across the seven sequencing

platforms (Fig. S2 and Table S1). Based on the filtering criteria, the newest platforms, NovaSeq6000 and DNBSEQ-T7, showed the lowest percentage of low-quality reads (2.8% and 4.2%, respectively).

We analyzed the frequency of random sequencing errors (ambiguous base, N), which is also an important factor to evaluate the quality of the sequencing platform. We found that the HiSeq2000, HiSeq4000, and HiSeqX10 showed a high random error ratio in certain sequencing cycles (Fig. S3 and Table S2). Furthermore, in the case of HiSeq2000, the random error tended to increase gradually after each sequencing cycle. We also investigated the sequencing error using *K*-mer analysis. Most erroneous *K*-mers caused by sequencing error appeared at very low frequency and form a sharp left-side peak [13, 14]. Distribution of *K*-mer frequencies showed similar distributions between the platforms (Fig. 1). However, there was a difference in the proportion of low-frequency *K*-mer (≤ 3 *K*-mer depth), which was considered as putative sequencing errors (Table S3). The NovaSeq6000 showed the lowest amount of erroneous *K*-mer (3.91%), while the HiSeq4000 contained the highest amount of erroneous K-mer (13.91%) among the seven sequencing platforms. The BGISEQ-500 and DNBSEQ-T7 showed a moderate level of erroneous *K*-mer (7.72% and 6.39%, respectively).

We examined the duplication rate and adapter contamination in the seven sequencing platforms (Table S2). We examined the exact duplicates, which are identical sequence copies, from raw sequence data. The HiSeq2000 and DNBSEQ-T7 showed the highest duplicate ratio (8.71% in HiSeq2000 and 3.04% in DNBSEQ-T7). The HiSeq4000, HiSeqX10 and NovaSeq6000 showed higher adapter contamination rates than other platforms, probably due to longer sequence length (151 bp). However, duplicates and adapter contamination may be more affected by the process of sample preparation than by the sequencing instrument.

Genome coverage and sequencing uniformity

In order to assess genomic coverage and sequencing uniformity, we aligned quality-filtered reads to the human reference genome (GRCh38). All seven sequencing platforms showed a mapping rate of more than 99.98% and genome coverage of more than 99.6% (≥ 1×; Table 2). We observed a higher duplicate mapping rate in the HiSeq2000 (15.35%) and DNBSEQ-T7 (8.77%) than the other platforms and the same pattern as the duplication rates of raw reads (see Table S2). Additionally, it was also observed that duplication rates of other DNBSEQ-T7 data were also high, which were generated by the same run with the KOREF data (Table S4). The insert-size for paired-end libraries corresponds to the targeted fragment size for each platform (Fig. S4). It has been reported that the depth of coverage is often far from evenly distributed across the sequenced genome [15]. To assess the sequencing uniformity, we analyzed the distribution of mapping depth for all chromosomes (Fig. S5). All seven platforms showed a similar pattern of depth distribution, but interestingly, we found that the depth near the centromere regions was lower exclusively in the HiSeq4000 (Figs. S6-S9). We speculate that this may have been due to a bias in the library preparation step on the HiSeq4000 platform.

In order to examine the platform-specific covered region of MGI and Illumina platforms, we defined a platform-specific covered region that had significantly different depths based on the 100 bp non-overlapping windows and statistical test [16]. Prior to examining the platform-specific covered regions, mapped reads were down-sampled for all platforms to 24× coverage, which is the minimum coverage among the platforms, for a fair comparison. (Table S5). We found 178 Kb and 297 Kb of the platform-specific covered regions from MGI and Illumina platforms, respectively (Table S6). A total of 168 and 373 genes were overlapped in MGI and Illumina specific covered regions, respectively, and most of them were intronic. Interestingly, however, the platform-specific covered regions showed a significantly different

distribution of GC ratios between the MGI and Illumina platforms (Fig. S10). The MGI platforms tend to cover regions relatively high in GC content (Wilcoxon rank-sum test, $P = 2.37 \times 10^{-133}$). Nevertheless, it is obvious that platform-specific covered regions for Illumina platforms are slightly longer than those of the MGI platforms, and these regions were not sufficiently covered by the MGI platforms.

Biases in PCR amplification create uneven genomic representation in classical Illumina libraries [17, 18] as PCR is sensitive to extreme GC-content variation [19]. Thus, we analyzed the GC biases for seven sequencing platforms. We examined the distribution of GC content in sequencing reads and found that raw reads of all the seven sequencing platforms showed a similar GC content distribution to the human reference genome (Fig. S11). To better understand what parts of the genome were not covered properly, we generated GC-bias plots, showing relative coverage at each GC level. Unbiased sequencing would not be affected by GC composition, resulting in a flat line along with relative coverage = 1. We found that all the seven sequencing platforms provided nearly even coverage in the moderate-GC range 20% to 60%, which represents approximately 95% of the human genome (Fig. 2). On the other hand, the relative coverage of the HiSeq2000 platform dropped fast above 60% GC than other platforms, while the NovaSeq6000 covered well above 60% GC, unlike the other platforms.

Comparison of variants detected among seven sequencing platforms

To investigate the performance of variant calling for the seven sequencing sequencers, we adopted the widely used pipeline BWA-MEM (BWA, RRID:SCR_010910) [20] and GATK (GATK, RRID:SCR_001876) [21-23]. We identified an average of 4.14 million single nucleotide variants (SNVs), and 0.61 million indels (insertion and deletion) on each of the

seven sequencing platforms (Table 3). The statistics of SNVs were similar across all the seven in terms of the dbSNP annotation rate (dbSNP153) and the transition/transversion (Ti/Tv) ratio, which indirectly reflects SNV calling accuracy. About 3.7 million SNV loci were found on all the seven sequencing platforms, and this accounts for 87% to 91% of the discovered SNVs on each platform (Table S7). We found 13,999 and 9,691 platform-specific SNVs on the MGI and Illumina platforms, respectively. To figure out the potential cause of the platform-specific SNVs, we checked how many of the SNVs were located on the platform-specifically covered regions. There were only 2.8% of Illumina platform-specific SNVs and 1.6% of MGI platform-specific SNVs that were located on the platform-specifically covered region (Table S8), and most of the platform-specific SNVs were located on regions with sufficient sequencing depths (>10×). It was also found that about 74% of platform-specific SNVs were located on the repeat region (Table S9). The number of singletons, variations found only in one platform, was higher for the Illumina (~0.10 million SNVs on average) than MGI (~0.05 million SNVs on average) sequencers (see Table S7). This means that the difference within the Illumina platforms is greater than the difference between the MGI platforms. Similar to the case of the platform-specific SNVs, a few singletons were found in the platform-specific covered region (0.5% in average), and most of the singletons were located on sufficiently high sequencing depth regions (>10×, Table S10). About 74% of singletons were located on the repeat region (see Table S9). We speculate that the repeat region is one of the sources causing the platform-specific SNVs and singletons. We also analyzed the number of SNVs found in any six of the seven sequencing platforms, which we considered false negatives (Table S11). The HiSeq2000 had the largest number of false negatives (64,856 SNVs) among the seven sequencing platforms. The two MGI platforms (DNBSEQ-T7 and BGISEQ-500) had 18,826 and 15,657 false negatives, respectively, and those of the NovaSeq6000 showed the smallest number of false negatives (6,999 SNVs). To investigate the relationship between the sequencing platforms, an unrooted

tree was constructed using a total of 1,036,417 loci where the genotypes of one or more platforms differ from the rest of the platforms (Fig. 3 and Table S12). We found that the two MGI platforms grouped together, and they are the closest to the Illumina HiSeq2500 platform. The Illumina platforms were divided into two subgroups in the tree: a long read length (151 bp) group, containing the HiSeq4000, HiSeqX10, and NovaSeq6000 platforms and a short read length (≤101 bp) group, containing the HiSeq2000 and HiSeq2500 platforms. Read length primarily affects the detection of variants through alignment bias and alignment errors, which are higher for short reads because there is less chance of a unique alignment to the reference sequence than with longer reads [24].

Since it was not possible to conduct standard benchmarking procedures and determine error values for each platform in this study, we compared the variations called by the seven whole-genome sequences with an SNP genotyping chip as an independent platform. Of the total 950,585 comparable positions, more than 99.3% of the genotypes matched the WGS-based genotypes from the seven sequencing platforms (Table S13). We found that 4,356 loci in the SNP genotyping were inconsistent across all seven WGS-based genotyping results, suggesting that these loci are probably errors in the SNP genotyping chip. With the exception of HiSeq2000 and HiSeq4000, all the other platforms showed a similar concordance rate.

Discussion

Our benchmarks provided here can provide a useful but rough estimation of the quality of short-read based whole-genome sequencers. We used the same individual's samples for all seven sequencing platforms but these were collected at different time points over the past seven years. Just one human sample cannot justify the variation that may occur among different

individuals, extracted DNA molecules, and overall sequencing qualities. Furthermore, the sequencing quality may vary greatly depending on the version of the library preparation kit, even on the same platform [25]. These are clear limitations of our benchmarking, however, as our purpose was to compare two major platforms, namely Illumina and MGI, the whole genome data from just one individual can function as an intuitive index for researchers who are considering purchasing large sequencers to generate a very large amount of sequencing data (Table S14). Our method of statistical analysis does not allow us to conclude which of the seven sequencing instruments is the most accurate and precise as there is much variation in the sample preparation and sequencer specifications. Nevertheless, overall, the data generated by the Illumina and MGI sequencing platforms showed comparable levels of quality, sequencing uniformity, %GC coverage, and concordance rate with SNP genotyping, thus it can be broadly concluded that the MGI platforms can be used for a wide range of research tasks on a par with Illumina platforms, and at a lower cost [7].

Materials and Methods

Genomic DNA extraction and SNP genotyping

Genomic DNA used for genotyping and sequencing were extracted from the peripheral blood of a Korean male sample donor (KOREF). The genomic DNA was extracted using the DNeasy Blood & Tissue kit (Qiagen, Valencia, CA) according to the manufacturer's recommendations. DNA quality was assessed by running 1 μl on the Bioanalyzer system (Agilent) to ensure size and analysis of DNA fragments. The concentration of DNA was assessed using the dsDNA BR assay on a Qubit fluorometer (Thermo Fisher). We conducted a genotyping experiment using the Illumina Infinium Omni1 quad chip according to the manufacturer's protocols. The Institutional Review Board (IRB) at Ulsan National Institute of Science and Technology approved the study (UNISTIRB-15-19-A).

Illumina paired-end library construction and sequencing

High-molecular weight genomic DNA was sheared using a Covaris S2 ultra sonicator system, in order to get appropriate sizes. Libraries with short inserts of 500 bp for HiSeq2000, 400 bp for HiSeq2500 (RRID:SCR_016383) and HiSeq4000 (RRID:SCR_016386), and 450 bp for HiSeqX10 and NovaSeq6000 for paired-end reads were prepared using TruSeq DNA sample prep kit following the manufacturer's protocol. Products were quantified using the Bioanalyzer (Agilent, Santa Clara, CA, USA) and the raw data were generated by each Illumina platform. Further image analysis and base calling were conducted with the Illumina pipeline using default settings.

MGI paired-end library construction and sequencing

The KOREF genomic DNA was fragmented by Frag enzyme (MGI) to DNA fragments between 100 bp and ~1,000 bp suitable for PE100 sequencing according to the manufacturer's instructions (MGI FS DNA library prep set, cat no; 1000005256). The fragmented DNA was further selected to be between 300 bp and ~500 bp by DNA clean beads (MGI). The selected DNA fragments were then repaired to obtain a blunt end and modified at the 3'end to get a dATP as a sticky end. The dTTP tailed adapter sequence was ligated to both ends of the DNA fragments. The ligation product was then amplified for seven cycles and subjected to the following single-strand circularization process. The PCR product was heat-denatured together with a special molecule that was reverse-complemented to one special strand of the PCR product, and the single-strand molecule was ligated using DNA ligase. The remaining linear molecule was digested with the exonuclease, finally obtaining a single-strand circular DNA library. We sequenced the DNA library using BGISEQ-500 (RRID:SCR_017979) and DNBSEQ-T7 (RRID:SCR_017981) with a pair-end read length of 100bp.

Raw data preprocessing

We used the FastQC v0.11.8 (FastQC, RRID:SCR_014583) [12] to assess overall sequencing quality for MGI and Illumina sequencing platforms. PCR duplications (reads were considered duplicates when forward read and reverse read of the two paired-end reads were identical) were detected by the PRINSEQ v0.20.4 (PRINSEQ, RRID:SCR_005454) [26]. The random sequencing error rate was calculated by measuring the occurrence of 'N' bases at each read position in raw reads. Reads with sequencing adapter contamination were examined according

to the manufacturer's adapter sequences (Illumina sequencing adapter left = "*GATCGGAAGAGCACACGTCTGAACTCCAGTCAC*", Illumina sequencing adapter right = "*GATCGGAAGAGCGTCGTGTAGGGAAAGAGTGT*", MGI sequencing adapter left = "*AAGTCGGAGGCCAAGCGGTCTTAGGAAGACAA*", and MGI sequencing adapter right = "*AAGTCGGATCGTAGCCATGTCGTTCTGTGAGCCAAGGAGTTG*"). We conducted base quality filtration of raw reads using the NGS QC Toolkit v2.3.3 (cutoff read length for high quality 70; cutoff quality score, 20) (NGS QC Toolkit, RRID:SCR_005461) [27]. We used clean reads after removing low-quality reads and adapter containing reads for the mapping step.

Mapping, variant calling, and coverage calculation

After the filtering step, clean reads were aligned to the human reference genome (GRCh38) using BWA-MEM v0.7.12, and duplicate reads were removed using Picard v2.6.0 (Picard, RRID:SCR_006525) [28]. After removing duplicate reads, we down-sampled the deduplicated clean reads of all the sequencing platforms to 24× coverage according to the amount of the deduplicated clean reads of HiSeq2000 for a fair comparison. Realignment and base score recalibration of the bam file was processed by GATK v3.3. Single nucleotide variants, short insertions, and deletions were called with the GATK (Unifiedgenotyper, options --output_mode EMIT_ALL_SITES --genotype_likelihoods_model BOTH). The resulting variants were annotated with the dbSNP (v153) database [29]. Coverage was calculated for each nucleotide using SAMtools v1.9 (SAMTOOLS, RRID:SCR_002105) [30]. We defined a specific covered region based on the 100 bp non-overlapping windows by calculating the average depth of the windows followed by a statistical test. We used edgeR method as the statistical test [16]. *P*-values are adjusted by Benjamini-Hochberg correction. GC coverage for

raw reads and the genome was calculated by the average %GC of the 100bp non-overlapping windows.

Variant comparison and concordance rate with SNP genotyping

The chromosome position and genotype of each variant called from each sequencing platform was used to identify the relationship between seven sequencing platforms. We compared 1,036,417 loci found on one or more platforms for locations where genotypes were determined on all the seven platforms. An unrooted tree was generated using FastTree v2.1.10 (FastTree, RRID:SCR_015501) [31] with the generalized time-reversible (GTR) model. For calculating the concordance rate between SNP genotyping and WGS-based genotype, the coordinates of SNP genotyping data were converted to GRCh38 assembly using the UCSC LiftOver tool [32]. We removed unmapped positions and indel markers and used only markers that were present on the autosomal chromosomes.

Availability of Supporting Data and Materials

All sequences generated in this study, including the HiSeq2000, HiSeq2500, HiSeq4000, HiSeqX10, NovaSeq6000, BGISEQ-500, and DNBSEQ-T7 sequencing reads, were deposited in the NCBI Sequence Read Archive database under BioProject PRJNA600063. All benchmarking data is hosted and distributed from the biosequencer.org homepage [33], and supporting data and materials are also available at *GigaScience* GigaDB [34].

Additional Files

Additional file 1: **Figure S1**. Distribution of nucleotide quality across seven sequencing platforms. **Figure S2**. Base quality filtration statistics for seven sequencing platforms. **Figure S3**. Random error ratio for seven sequencing platforms. **Figure S4**. Insert-size distributions for seven sequencing platforms. **Figure S5**. The coverage distribution of two MGI and five Illumina platforms. **Figure S6**. Depth distribution of chromosome 8. **Figure S7**. Depth distribution of chromosome 12. **Figure S8**. Depth distribution of chromosome 18. **Figure S9**. Depth distribution of chromosome 20. **Figure S10**. GC distribution of platform-specific covered regions. **Figure S11**. The GC composition distribution of the human genome and sequencing reads. **Table S1**. Base quality summary. **Table S2**. Duplicate reads, random error base, and adapter read rate. **Table S3**. The putatively erroneous *K*-mers (≤ 3 *K*-mer depth) for seven sequencing platforms. **Table S4**. Mapping and duplicate rate of samples using MGI's PE100 protocol and DNBSEQ-T7. **Table S5**. Statistics of clean reads for seven sequencing platforms. **Table S6**. Statistics for platform-specific covered regions. **Table S7**. The number of shared SNVs for seven sequencing platforms. **Table S8.** Statistics of platform-specific SNVs. **Table S9.** Statistics of platform-specific SNVs and singleton in the repeat region. **Table S10.** Statistics of singleton variants. **Table S11**. The number of SNVs not found on a specific platform. **Table S12**. Genotype concordance rate among seven sequencing platforms. **Table S13**. Genotype comparison between SNP genotyping and WGS. **Table S14**. Recent studies for MGI and Illumina platform comparison.

List of abbreviations

PE: paired-end;

WGS: whole-genome sequencing;

BWA: burrows-wheeler aligner;

SNVs: single nucleotide variants;

indels: insertions and deletions;

Ti/Tv: transition/transversion;

GATK: Genome Analysis ToolKit;

Competing Interests

H.M.K., O.C., Y.S.C., J.H.J., H.Y.L., and Y.Y. are employees, J.B. is the chief executive officer of Clinomics Inc. H.M.K., Y.S.C., and J.B. have an equity interest in the company. All other co-authors declare that they have no competing interests.

Funding

This work was supported by the Research Project Funded by the Ulsan City Research Fund (1.200047.01) of the Ulsan National Institute of Science & Technology (UNIST) and Clinomics and Geromics Ltd internal funding. This work was also supported by the Technology Innovation Program (20010587, Development and Dissemination on National

Standard Reference Data) funded by the Ministry of Trade, Industry & Energy (MOTIE, Korea).

Authors' contributions

J.B. supervised and coordinated the project. J.B. and Y.S.C. conceived and designed the experiments. H.M.K., S.J., O.C., J.H.J., H.Y.L., and Y.Y. conducted the bioinformatics data processing and analyses. H.M.K., S.J., D.M.B., and J.B. wrote and revised the manuscript. A.B. and H.S.K. reviewed and edited the manuscript. All authors have read and approved the final manuscript.

Acknowledgments

We thank the Korea Institute of Science and Technology Information (KISTI) for providing us with the Korea Research Environment Open NETwork (KREONET). We thank Jaesu Bhak for editing.

References

1. Wetterstrand K. DNA sequencing costs: data: data from the NHGRI Genome Sequencing Program (GSP). 2018, https://www.genome.gov/about-genomics/fact-sheets/DNA-Sequencing-Costs-Data (12 March 2020, date last accessed)
2. Huddleston J, Chaisson MJP, Steinberg KM, et al. Discovery and genotyping of structural variation from long-read haploid genome sequence data. Genome Res 2017;**27**(5):677-85.
3. Goodwin S, McPherson JD and McCombie WR. Coming of age: ten years of next-generation sequencing technologies. Nat Rev Genet 2016;**17**(6):333-51.
4. Drmanac R, Sparks AB, Callow MJ, et al. Human genome sequencing using unchained base reads on self-assembling DNA nanoarrays. Science 2010;**327**(5961):78-81.
5. Huang J, Liang X, Xuan Y, et al. A reference human genome dataset of the BGISEQ-500 sequencer. Gigascience 2017;6(5):1-9.
6. Chen J, Li X, Zhong H, et al. Systematic comparison of germline variant calling pipelines cross multiple next-generation sequencers. Sci Rep. 2019;9 1:9345. doi:10.1038/s41598-019-45835-3.
7. Jeon SA, Park JL, Kim JH, et al. Comparison of the MGISEQ-2000 and Illumina HiSeq 4000 sequencing platforms for RNA sequencing. Genomics Inform. 2019;17 3:e32. doi:10.5808/GI.2019.17.3.e32.
8. Fang C, Zhong H, Lin Y, et al. Assessment of the cPAS-based BGISEQ-500 platform for metagenomic sequencing. Gigascience. 2018;7 3:1-8. doi:10.1093/gigascience/gix133.
9. Lu R, Zhao X, Li J, et al. Genomic characterisation and epidemiology of 2019 novel coronavirus: implications for virus origins and receptor binding. Lancet. 2020;395

10224:565-74. doi:10.1016/S0140-6736(20)30251-8.

10. Kim D, Lee JY, Yang JS, et al. The Architecture of SARS-CoV-2 Transcriptome. Cell. 2020;181 4:914-21 e10. doi:10.1016/j.cell.2020.04.011.

11. Cho YS, Kim H, Kim HM, et al. An ethnically relevant consensus Korean reference genome is a step towards personal reference genomes. Nat Commun. 2016;7:13637. doi:10.1038/ncomms13637.

12. Andrews S, Krueger F, Segonds-Pichon A, et al. FastQC. Babraham. 2012. https://www.bioinformatics.babraham.ac.uk/projects/fastqc/. Accessed 08 February 2021.

13. Zhao L, Xie J, Bai L, et al. Mining statistically-solid k-mers for accurate NGS error correction. BMC Genomics. 2018;19 Suppl 10:912. doi:10.1186/s12864-018-5272-y.

14. Liu B, Shi Y, Yuan J, et al. Estimation of genomic characteristics by analyzing k-mer frequency in de novo genome projects. arXiv preprint arXiv:13082012. 2013.

15. Dohm JC, Lottaz C, Borodina T, et al. Substantial biases in ultra-short read data sets from high-throughput DNA sequencing. Nucleic Acids Res. 2008;36 16:e105. doi:10.1093/nar/gkn425.

16. Robinson MD, McCarthy DJ, Smyth GK. edgeR: a Bioconductor package for differential expression analysis of digital gene expression data. Bioinformatics. 2010;26 1:139-140. doi:10.1093/bioinformatics/btp616.

17. Kozarewa I, Ning Z, Quail MA, et al. Amplification-free Illumina sequencing-library preparation facilitates improved mapping and assembly of (G+C)-biased genomes. Nat Methods. 2009;6 4:291-5. doi:10.1038/nmeth.1311.

18. Aird D, Ross MG, Chen WS, et al. Analyzing and minimizing PCR amplification bias in Illumina sequencing libraries. Genome Biol. 2011;12 2:R18. doi:10.1186/gb-2011-12-2-r18.

19. Oyola SO, Otto TD, Gu Y, et al. Optimizing Illumina next-generation sequencing library preparation for extremely AT-biased genomes. BMC Genomics. 2012;13:1. doi:10.1186/1471-2164-13-1.

20. Li H. Aligning sequence reads, clone sequences and assembly contigs with BWA-MEM. arXiv preprint arXiv:13033997. 2013.

21. DePristo MA, Banks E, Poplin R, et al. A framework for variation discovery and genotyping using next-generation DNA sequencing data. Nat Genet. 2011;43 5:491-8. doi:10.1038/ng.806.

22. Van der Auwera GA, Carneiro MO, Hartl C, et al. From FastQ data to high confidence variant calls: the Genome Analysis Toolkit best practices pipeline. Curr Protoc Bioinformatics. 2013;43:11 0 1- 0 33. doi:10.1002/0471250953.bi1110s43.

23. McKenna A, Hanna M, Banks E, et al. The Genome Analysis Toolkit: a MapReduce framework for analyzing next-generation DNA sequencing data. Genome Res. 2010;20 9:1297-303. doi:10.1101/gr.107524.110.

24. Patch AM, Nones K, Kazakoff SH, et al. Germline and somatic variant identification using BGISEQ-500 and HiSeq X Ten whole genome sequencing. PLoS One. 2018;13 1:e0190264. doi:10.1371/journal.pone.0190264.

25. Rhodes J, Beale MA and Fisher MC. Illuminating choices for library prep: a comparison of library preparation methods for whole genome sequencing of Cryptococcus neoformans using Illumina HiSeq. PLoS One. 2014;9 11:e113501. doi:10.1371/journal.pone.0113501.

26. Schmieder R and Edwards R. Quality control and preprocessing of metagenomic datasets. Bioinformatics. 2011;27 6:863-4. doi:10.1093/bioinformatics/btr026.

27. Patel RK and Jain M. NGS QC Toolkit: a toolkit for quality control of next generation sequencing data. PLoS One. 2012;7 2:e30619. doi:10.1371/journal.pone.0030619.

28. Institute B: Picard: A set of command line tools (in Java) for manipulating high-throughput sequencing (HTS) data and formats such as SAM/BAM/CRAM and VCF. GitHub 2018. http://broadinstitute.github.io/picard/.

29. Sherry ST, Ward MH, Kholodov M, et al. dbSNP: the NCBI database of genetic variation. Nucleic Acids Res. 2001;29 1:308-11. doi:10.1093/nar/29.1.308.

30. Li H, Handsaker B, Wysoker A, et al. The Sequence Alignment/Map format and SAMtools. Bioinformatics. 2009;25 16:2078-9. doi:10.1093/bioinformatics/btp352.

31. Price MN, Dehal PS and Arkin AP. FastTree 2--approximately maximum-likelihood trees for large alignments. PLoS One. 2010;5 3:e9490. doi:10.1371/journal.pone.0009490.

32. Kuhn RM, Haussler D and Kent WJ. The UCSC genome browser and associated tools. Brief Bioinform. 2013;14 2:144-61. doi:10.1093/bib/bbs038.

33. BioSequencer, http://biosequencer.org. Accessed 08 February 2021.

34. Kim H, Jeon S, Chung O, et al. Supporting data for "Comparative analysis of seven sh ort-reads sequencing platforms using the Korean Reference Genome: MGI and Illumi na sequencing benchmark for whole-genome sequencing" *GigaScience* Database 2021 http://dx.doi.org/10.5524/100865.

Figures

Figure 1. Distribution of *K*-mer frequency for 21-mers using raw reads from seven sequencing platforms. The x-axis represents *K*-mer depth, and the y-axis represents the proportion of *K*-mer, as calculated by the frequency at that depth divided by the total frequency at all depths.

Figure 2. GC-bias plots for seven sequencing platforms. Unbiased coverage is represented by a horizontal dashed line at relative coverage = 1. A relative coverage below 1 indicates lower than expected coverage and above 1 indicates higher than expected coverage.

Figure 3. An unrooted tree for seven sequencing platforms showing the similarity of the variant calling. Numbers of nodes denote bootstrap values based on 1,000 replicates.

Tables

Table 1. Raw read statistics for seven sequencing platforms

	Illumina platforms					MGI platforms	
Metrics	HiSeq2000	HiSeq2500	HiSeq4000	HiSeqX10	NovaSeq6000	BGISEQ-500	DNBSEQ-T7
Production date	2012	2015.03	2015.10	2015.12	2019.04	2017.04	2019.09
Quality range	Illumina 1.5+	Illumina 1.8+	Illumina 1.8+	Illumina 1.8+	Illumina 1.8+	Illumina 1.8+	Illumina 1.8+
# of Total read	1,044M	1,500M	629M	833M	833M	1,171M	1,035M
Read length (bp)	90 PE	101 PE	151 PE	151 PE	151 PE	100 PE	100 PE
Total bases	94 Gb	151.5 Gb	95 Gb	125.8 Gb	125.8 Gb	117.1 Gb	103.4 Gb
Sequencing depth (×, based on 3 Gb)	31.31	50.52	31.65	41.94	41.94	39.04	34.49

Table 2. Mapping and coverage statistics

Metrics	HiSeq2000	HiSeq2500	HiSeq4000	HiSeqX10	NovaSeq6000	BGISEQ-500	DNBSEQ-T7
# of clean reads	935,951,974	1,050,028,628	512,891,970	705,987,420	706,000,000	1,060,837,856	991,021,996
Read length	90	101	151	151	151	100	100
Clean bases (Gb)	84.23	106.05	77.45	106.60	106.6	106.08	99.1
Clean read depth (based on 3 Gb, ×)	28.08	35.35	25.82	35.53	35.54	35.36	33.03
Mapping rate	99.986%	99.999%	99.990%	99.999%	99.9996%	99.983%	99.999%
Properly mapped rate*	96.67%	98.30%	97.24%	96.91%	97.15%	97.44%	98.17%
Duplicate rate	15.35%	3.01%	3.19%	5.08%	3.39%	2.56%	8.77%
Duplicate clean read depth (×)	23.90	34.29	24.99	33.73	34.33	34.46	30.14
Down-sampled depth (×)	23.90	23.90	23.90	23.90	23.90	23.90	23.90
Coverage	99.68%	99.82%	99.71%	99.81%	99.76%	99.83%	99.83%
Coverage at least 5×	98.62%	99.30%	98.37%	99.30%	99.19%	99.34%	99.24%
Coverage at least 10×	94.63%	96.65%	93.98%	97.05%	96.89%	97.05%	96.61%
Coverage at least 15×	85.10%	88.54%	85.08%	90.23%	90.36%	90.11%	89.36%

* Both of the read mates are in the correct orientation.

Table 3. Variant statistics of Illumina and MGI sequencing platforms.

Metrics		HiSeq2000	HiSeq2500	HiSeq4000	HiSeqX10	NovaSeq6000	BGISEQ-500	DNBSEQ-T7
Reference homozygous		2,839,358,003	2,855,619,759	2,855,062,233	2,864,272,103	2,861,198,782	2,851,898,568	2,853,066,635
# of no call positions		80,241,142	63,980,549	64,532,078	55,244,498	58,311,103	67,747,107	66,584,361
No call rate		2.74%	2.19%	2.21%	1.89%	1.99%	2.32%	2.28%
SNVs	Total SNVs	4,133,925	4,132,468	4,138,296	4,216,589	4,223,612	4,088,645	4,082,103
	Total SNVs in dbSNP	4,094,212	4,114,993	4,112,253	4,198,005	4,184,100	4,070,101	4,064,986
	dbSNP rate	99.04%	99.58%	99.37%	99.56%	99.06%	99.55%	99.58%
	Singleton	159,429	78,109	98,574	100,158	104,052	52,127	51,978
	Singleton in dbSNP	126,762	68,673	78,361	89,094	73,177	41,092	41,743
	dbSNP rate for Singleton	79.51%	87.92%	79.49%	88.95%	70.33%	78.83%	80.31%
	Homozygous	1,703,616	1,690,878	1,704,813	1,708,639	1,714,752	1,688,328	1,689,834
	Heterozygous	2,430,309	2,441,590	2,433,483	2,507,950	2,508,860	2,400,317	2,392,269
	Het/Hom ratio	1.43	1.44	1.43	1.47	1.46	1.42	1.42
	Ti/Tv ratio	1.91	1.92	1.9	1.88	1.85	1.92	1.92
Indels	Total Indels	526,504	546,918	491,899	689,357	708,062	703,873	631,163
	Total Indels in dbSNP	524,738	544,866	489,777	686,916	705,553	701,802	629,314
	dbSNP rate	99.66%	99.62%	99.57%	99.65%	99.65%	99.71%	99.71%
	Singleton	7,864	7,444	8,094	17,036	23,596	41,384	12,092
	Singleton in dbSNP	7,612	7,259	7,915	16,784	23,303	41,183	11,964
	dbSNP rate for Singleton	96.80%	97.51%	97.79%	98.52%	98.76%	99.51%	98.94%

Figure 1 Click here to access/download;Figure;Figure1.pdf

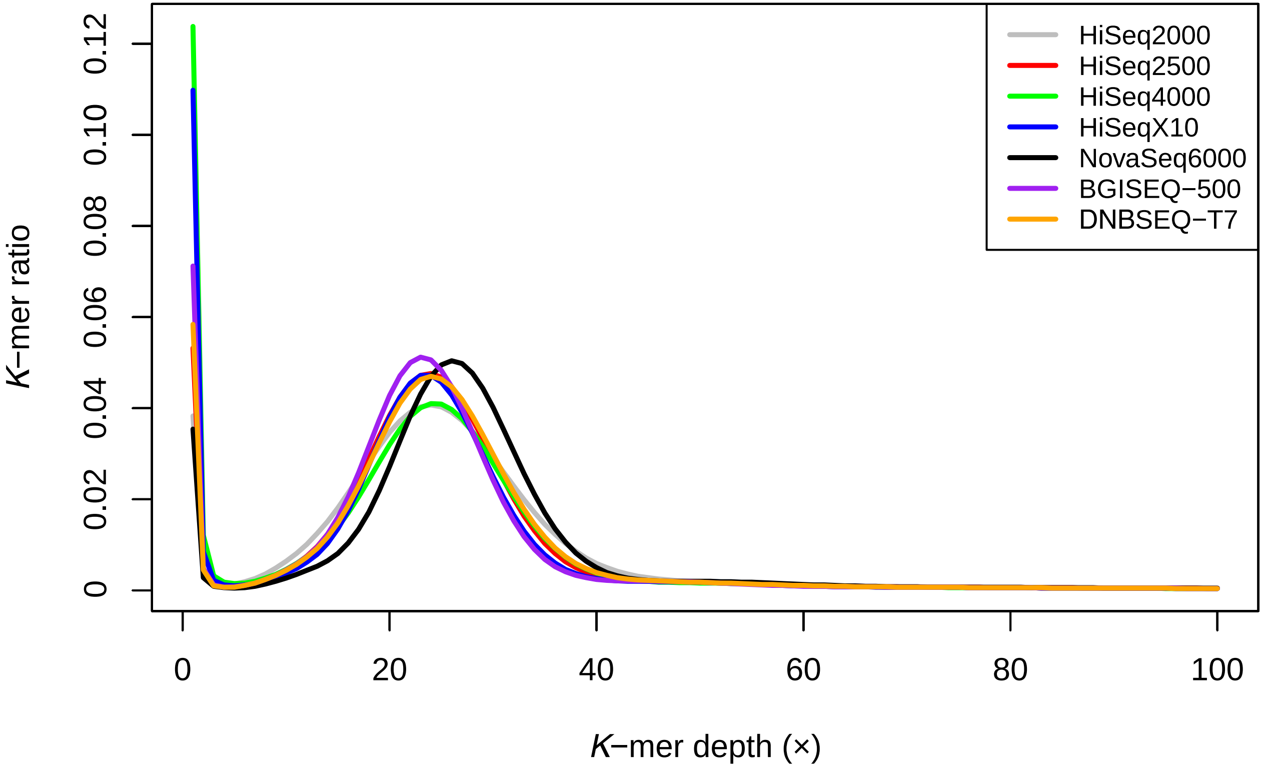

Figure 2

Click here to access/download;Figure;Figure2.pdf

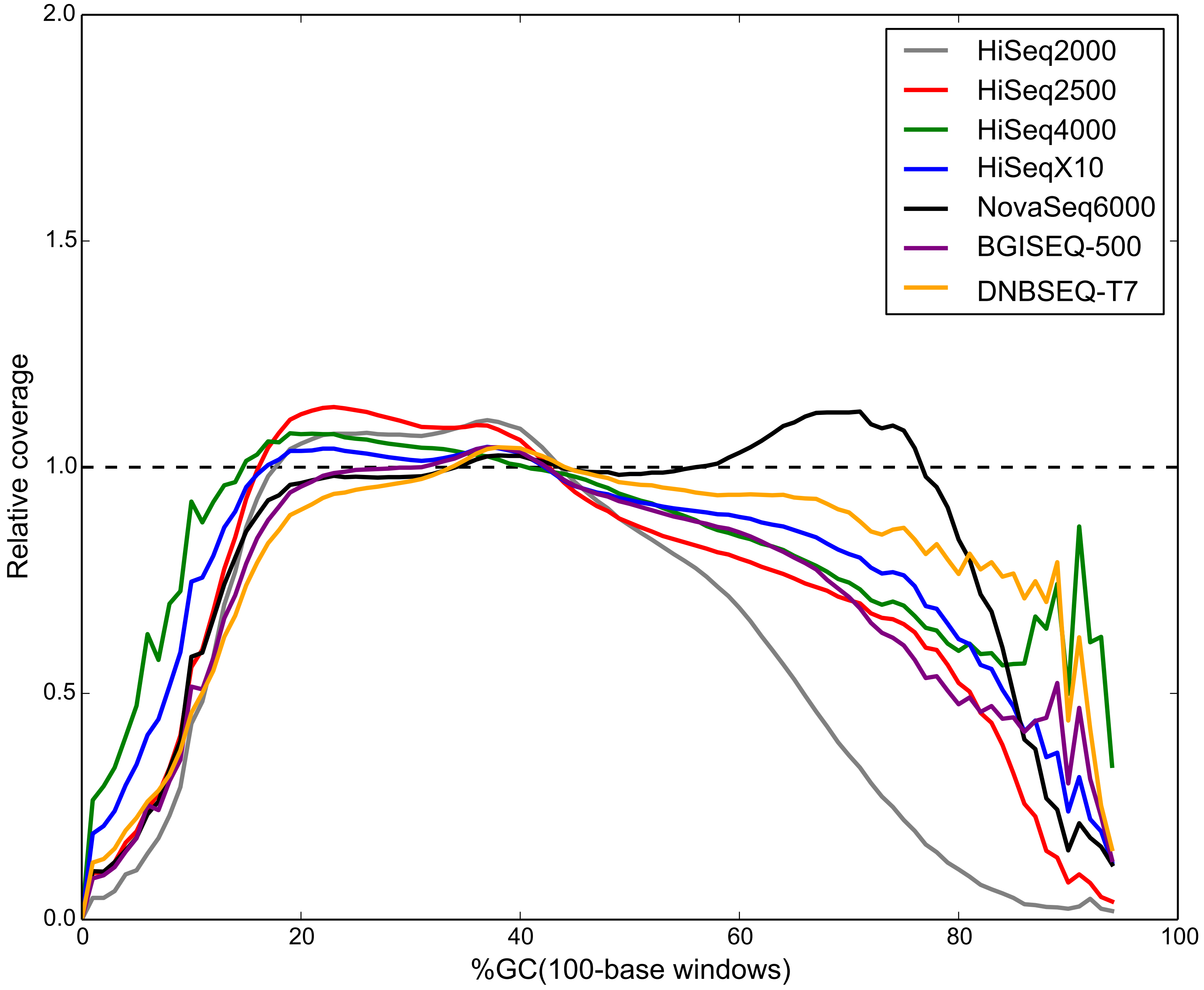

Figure 3

Click here to access/download;Figure;Figure3.PDF

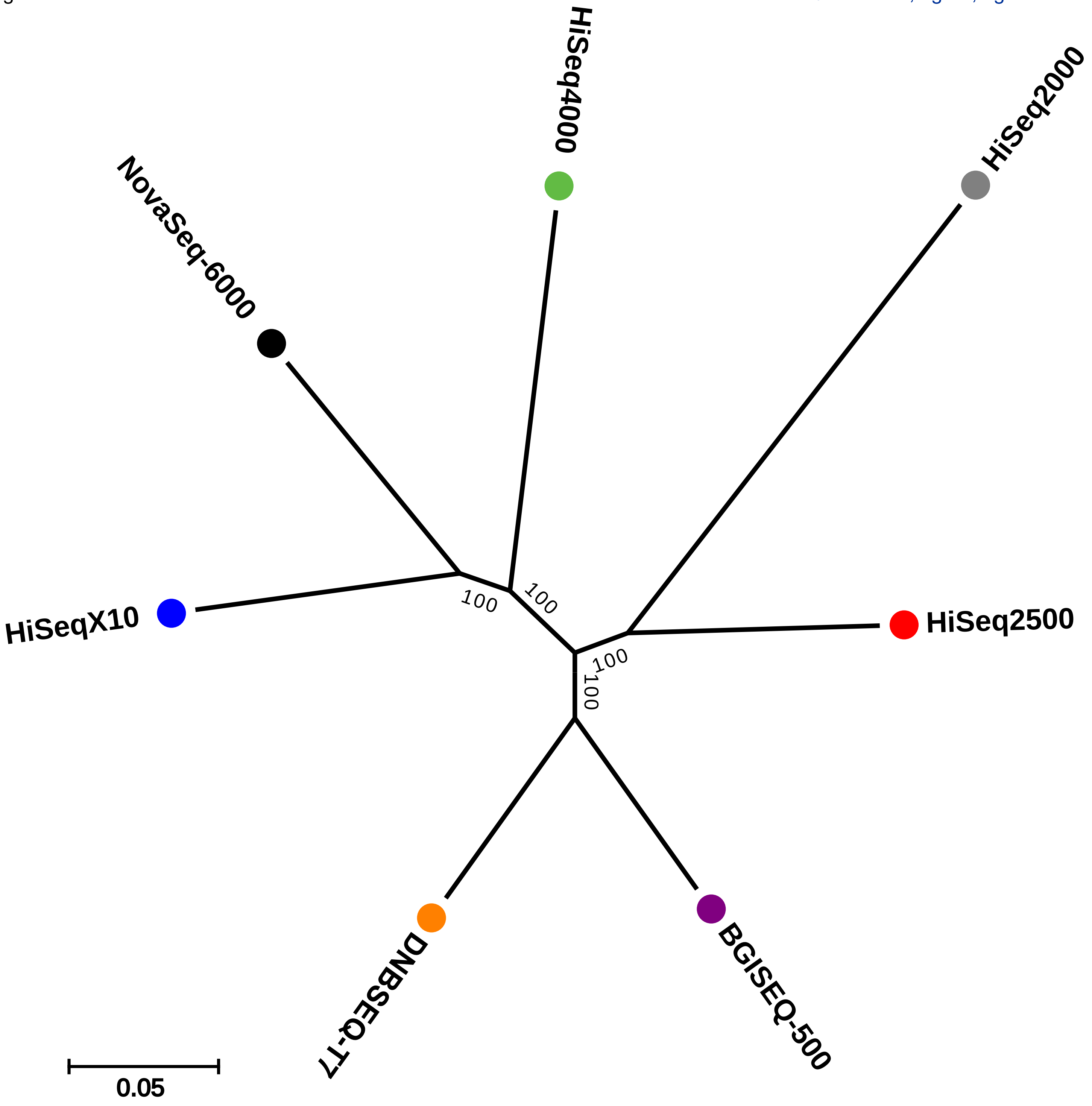

Response to reviewers

Click here to access/download
Supplementary Material
Sequencing_Platform_Comparison_RevisionNote_2nd_revision.docx

Personal Cover Click here to access/download;Personal Cover;Sequencing_Platform_Comparison_Coverletter_2nd_rev

GIGA-D-20-00072

Dear ***GigaScience*** editors,

Thank you very much for processing our manuscript for enhancing science and genomics.

We evaluated the reviewers' comments and have edited the manuscript to accommodate the reviewers' concerns on clarity and better presentation of our results. Please see the detailed point-by-point revision notes that are submitted on-line.

We hope that our revision will be suitable for your journal's standards.

Sincerely yours,

Jong Bhak, Ph.D.,
Professor, Biomedical Engineering,
KOGIC (Korean Genomics Center), UNIST, Ulsan, Korea
jongbhak@genomics.org

Dan M. Bolser, Ph.D.,
Director, Geromics Inc.
dan@geromics.co.uk;

29th Dec. 2020
