# Supplementary material for: Comparative analysis of 7 short-read sequencing platforms using the Korean Reference Genome: MGI and Illumina sequencing benchmark for whole-genome sequencing
Source: Gigascience. 2021 Mar 12;10(3):giab014. doi: 10.1093/gigascience/giab014 (PMC7953489; doi:10.1093/gigascience/giab014)
Supplement: giab014_Response_to_Reviewer_Comments_Original_Submission [file giab014_response_to_reviewer_comments_original_submission.pdf]

Author's Response To Reviewer Comments

Close

Reviewer reports:

Reviewer #1: In this manuscript, Kim et al. compared seven sequencing platforms, including 2 MGI platforms (BGISEQ-50
HiSeq4000, HiSeqX10, and NovaSeq6000), by using one human genome. The sequencing quality of different sequencing p
variant statistic. Overall the manuscript is suitable to be published on Giga Science after a major revision. There are sever
=> Thank you for precise and critical feedback. We have modified the text and added further analysis to accommodate th

1. This work only contains samples from one human individual. It's really hard to reach a confident conclusion based on su
=> It is a generally correct point. However, both platforms produce massive amounts of sequences and the sample numb
sets of platforms are similar or dissimilar in terms of variant calling.

This work still needs more samples and even replicates (both Cross-platform replicates and intra-platform replicates) to d
=> We think this is a practically important point. Unfortunately, we have not generated replicates for each sequencer. Firs
sample and each sequencing batch can contain multiple replicates or not. It is because each platform has a different amou
amount of sequences in a certain common replicate number. We stated these limitations in the discussion part of the man
platforms (MGI and Illumina).

2. The samples for sequencing were extracted on different points of time from the individual, that we wonder if the differe
different sampling time and the bias of sampling process.
=> There must be some problems caused by the different sampling time and the sampling process mentioned by the revi
and the last sampling time is about 7 years. It is known that the human germline mutation rate is approximately 0.5×10−
which means that 10.5 germline mutations can be accumulated in 7 years. In this respect, although the mutation rate of l
germline cell, the number of mutations accumulated over the 7 years would be much lower than the difference between p
significant effect on the results.
For the case of sampling process bias, we stated in the discussion part of the manuscript that there is a clear limitation in
mentioned, we think our study is still meaningful in that it provides the data generated by the short read-based whole ger
the long existing common Illumina platforms with the relatively new MGISEQ-T7 platform using one human whole genome

3. This manuscript needs to show more detail about the sequencing process, such as the number of the flow cell and sequ
each sequencing platform needs.
=> We added the detailed methods for DNA extraction, library preparation, and sequencing process in the Materials and M

4. In order to compare, the sequencing data of seven sequencing platforms need to have the same genome coverage.
=> Very good point. As pointed out by the reviewer, we set the same genome coverage of the seven platforms and updat
S5 and Table S4.

5. The results of the manuscript let me worry about the quality of the sequencing data generated from Hiseq2000 and His
the author found were normal.
=> HiSeq2000 and HiSeq4000 platforms are old, and their quality is not good compared to other platforms in our case. O
often not available in sequencing centers and, also, it is quite expensive to run them now. Still, to compare with MGI platf

6. According to the official information, MGI platforms have low duplicate rate than any sequencing platform which needs
the authors prove their finding by using other samples or individuals.
=> The official information showed a duplicate rate of less than 3% when using a PCR free library kit. However, we used t
duplicate rate is higher than the manufacturer's official information. We provide the table presenting the mapping rates ar
KOREF sample. We found that the duplicate rates of the other human samples that were sequenced simultaneously with t

https://github.com/howmany2/SequencingPlatformComparison/raw/master/Mapping%20and%20duplicate%20rate%20of

An FS library kit containing PCR steps was used for MGISEQ-T7 sequencing of the KOREF sample. Furthermore, according
high duplication rate, and the new PE150 (Paired-end 150 bp) protocol has a duplication rate less than 3%. We used the P
relatively many duplicated reads were found from the reads generated by the MGISEQ-T7 platform. However, we think th
after removing duplicate reads and matching to the same genome coverage for the seven sequencing platforms.

7. The methods for identifying the platform-specific covered region are unreasonable as different sequencing platforms ha
=> We agree with the reviewer's comment. We set the same genome coverage of the seven platforms and updated the re
platform decreased from 1,516 to 1,436, and in the case of Illumina, increased from 2,264 to 2,881. However, it was cont
as before meaning that the MGI platform covers a higher GC area (see Figure S10).

8. The Comparison of variants detected among seven platforms needs further analysis. Authors need a standard SNP and
sequencing or other methods, to replace the dbSNP and SNP genotype chip as a compare object. What the relationship of
=> We agree with the reviewer's comment that it is a powerful tool to compare the variants to the gold standard variant s
KOREF, which can give FP, FN, and sequencing error information, and, for this reason, we could not make a design for this
platforms. As an alternative, we examined how much difference exists among the sequences generated by different NGS p

9. The introduction of this manuscript is too simple.
=> We added several sequencing platform comparative studies to the introduction section.

Minor revisions:
1. The coverages of BGISEQ-500 and HiseqX10 were not mentioned in the first section.
=> We added the coverages of BGISEQ-500 and HiSeqX10 in the first section.

2. Using the ratio of singletons may help you to bring out your findings more clearly.
=> We agree with the reviewer's comment. We examined the concordance rate of the singleton variants with SNP genoty
below). However, it was difficult to obtain statistically significant results because there were very few overlapping position

https://github.com/howmany2/SequencingPlatformComparison/raw/master/Comparison%20between%20singleton%20va

Reviewer #2: The submitted study has characterized sequencing quality, uniformity of coverage, %GC coverage, and vari
showed a higher concordance rate of SNP genotyping than HiSeq series. The study is of interest to genomics and sequenc
acceptance.
=> Thank you for the feedback. We have modified the text and added further analysis to accommodate the reviewer's sug

1)The author defined low-quality reads as those that had more than 30% of bases with a sequencing quality score lower t
changed?
=> As a supplementary analysis, we conducted an analysis without the filtering step to see how much the read filtering st
conducted by matching the number of unfiltered reads with that of clean reads of prior analysis. The two tables below are
the cases using clean (filtered) and unfiltered sequences (see link below).

https://github.com/howmany2/SequencingPlatformComparison/raw/master/Mapping%20rate%20and%20Variant%20stat

As a result of using the unfiltered sequences, there was no notable difference in mapping and duplicate rates. The number
SNVs increased, the hetero/homo ratio increased by 0.02 on average. Interestingly, the differences in total SNVs between
the Illumina platforms. In the case of the Illumina platforms, on average, 44,000 additional SNVs were discovered when u
increment in MGI platform was 800 SNVs on average when using unfiltered reads.

2) It looks the author ignored a highest duplicate ratio was found in MGISEQ-T7. More discussion and analysis should be p
contamination may be more affected by the process of sample preparation than by the sequencing instrument. However, a
=> We agree with the reviewer's concerns about the high duplicate ratio. We provide the table presenting the mapping ra
with the KOREF sample. We found that the duplicate rates of other human samples that were sequenced simultaneously w
An FS library kit containing PCR steps was used for MGISEQ-T7 sequencing of the KOREF sample. Furthermore, according
high duplication rate, and the new PE150 (Paired-end 150 bp) protocol reduces the duplication rate to less than 3%. We u
reason why relatively many duplicated reads were found from the reads generated by the MGISEQ-T7 platform. However,
was analyzed after removing the duplicate reads and matching to the same genome coverage for the seven sequencing pl

https://github.com/howmany2/SequencingPlatformComparison/raw/master/Mapping%20and%20duplicate%20rate%20of

There are three main causes of duplicate reads generated by NGS technology.
1. Natural duplication
2. PCR duplicates (occur in library preparation step)
3. Optical duplicates (occur in sequencing step)
Natural duplications are not discussed in this section because it is difficult to distinguish them from PCR duplicates and opt

optical duplication of the seven platforms (see link below).

https://github.com/howmany2/SequencingPlatformComparison/raw/master/Statistics%20of%20PCR%20duplicate%20and

This result showed that PCR duplication occurs at least 2 times more than the optical duplication. (Unfortunately, the two
most duplication occurs during the library preparation rather than the sequencing steps.
The adapter contamination is caused by the sequencing of short DNA fragments that are shorter than the read length (Tur
expected that adapter contamination is mainly affected by the library preparation step, because size selection of DNA frag
selection can introduce the shorter DNA fragments into the DNA library for sequencing.

Reviewer #3: The authors compare various short-insert, short-read whole-genome sequencing platforms used by academ

My minor comments and suggestions are:

• As stated by the authors, Illumina platforms are indeed now considered 'historical.' However, many Illumina sequencers
prove very useful when arguing for an instrument upgrade in such a setting.

• You may like to comment on single tube long fragment read (stLFR), which enables the sequencing of long transcripts b
probably also MGISEQ-T7) (10.1101/gr.245126.118). This technology is relatively cheap and is likely to decrease in cost -

• You may want to comment on Illumina library kits. It is possible that revisions [in the five-six years since the data in yo
(e.g., see 10.1371/journal.pone.0113501). I realize the effect may be minor, but it may nevertheless be useful to remind
=> Thank you for your positive feedback and the suggestions. We added the idea suggested in your comments to the disc

Close
