# Supplementary material for: Comparative analysis of 7 short-read sequencing platforms using the Korean Reference Genome: MGI and Illumina sequencing benchmark for whole-genome sequencing
Source: Gigascience. 2021 Mar 12;10(3):giab014. doi: 10.1093/gigascience/giab014 (PMC7953489; doi:10.1093/gigascience/giab014)
Supplement: giab014_Response_to_Reviewer_Comments_Revision_1 [file giab014_response_to_reviewer_comments_revision_1.pdf]

Author's Response To Reviewer Comments

Close

Reviewer reports:

Reviewer #1: Much improved manuscript. I only have minor comments:
1) The examination of platform-specific covered region between MGI and Illumina platforms is still problematic. A single fold change threshold is unreliable. The authors should further make statistical test to identify platform-specific covered regions.
==> As pointed out by the reviewer, we re-analyzed the platform-specific covered region between MGI and Illumina platforms. We now use statistical test (edgeR method for group comparison followed by Benjamini-Hochberg correction for p-value adjustment) rather than the single fold change threshold to identify the platform-specifically covered region. As a result, the number of platform-specific covered regions of MGI platform increased from 1,436 to 1,778, and in the case of Illumina, increased from 2,881 to 2,967. We updated the manuscript and supplementary figure and table (See Results section lines 143-145; Figure S10 and Table S6).

2) Since the standard variant data set is not available, I think it is necessary to discuss the potential reason of the platform-specific SNVs and the singletons. Whether their distribution is associated with platform-specific covered regions or other reasons associated with low sequencing quality?
==> We speculate that repetitive regions with low mapping tendency were the one of the reasons for the platform-specific SNVs and singletons.
To figure out the potential reason of the platform-specific SNVs and the singletons, we compared these SNVs to platform-specific covered regions. First, we compared platform-specific SNVs to platform-specific covered regions. We found only 2.8% of Illumina platform-specific SNVs and 1.6% of MGI platform-specific SNVs are included in the platform specific covered region (Table S8). In addition, most of the platform-specific SNVs were located in a sufficient depth region (>10×), and about 74% of platform-specific SNVs were included in the repeat region (Table S9).
The singleton also showed a similar pattern to platform-specific SNVs. There were very few overlapping positions between the singleton variants and the platform-specific covered region (0.5% on average, Table S10), and most of the singletons were located in the relatively high depth region (>10×). About 74% of singletons were included in the repeat region (Table S9).
We updated these results to the manuscript (See Results section lines 179-194).

Reviewer #2: The authors addressed my and other reviewers's comments however many of the changes were quite minimal. It is suggested they can put the additional test in the main text and clarify all those limitations (not simple mentioned) in their study in the discussion section. For example, the high duplicate ratio in MGISEQ-T7 and a single individual was used.
==> Thanks for the comment. We now added additional result for platform-specific SNVs, singleton, and high duplicate ratio of MGISEQ-T7 platform in the manuscript (See Results section lines 134-135; Tables S4, S8, S9, and S10). Furthermore, we added a list of sequencing platform comparison studies using single individual in the discussion section (See Discussion section lines 221-228; Table S14).

Close
