# Supplementary material for: Comparative analysis of 7 short-read sequencing platforms using the Korean Reference Genome: MGI and Illumina sequencing benchmark for whole-genome sequencing
Source: Gigascience. 2021 Mar 12;10(3):giab014. doi: 10.1093/gigascience/giab014 (PMC7953489; doi:10.1093/gigascience/giab014)
Supplement: giab014_Reviewer_1_Report_Original_Submission — Dan Xie -- 5/11/2020 Reviewed [file giab014_reviewer_1_report_original_submission.pdf]

Reviewer Report

Title: Comparative analysis of seven short-read sequencing platforms using the Korean Reference Genome: MGI and Illumina sequencing benchmark for whole-genome sequencing

Version: Original Submission **Date:** 5/11/2020

Reviewer name: Dan Xie

Reviewer Comments to Author:

In this manuscript, Kim et al. compared seven sequencing platforms, including 2 MGI platforms (BGISEQ-500 and MGISEQ-T7) and 5 Illumina platforms (HiSeq2000, HiSeq2500, HiSeq4000, HiSeqX10, and NovaSeq6000), by using one human genome. The sequencing quality of different sequencing platform was assessed by basic sequencing statistics, mapping statistic and variant statistic. Overall the manuscript is suitable to be published on Giga Science after a major revision. There are several major issues with the work presented in the manuscript, as listed below:

1. This work only contains samples from one human individual. It's really hard to reach a confident conclusion based on such a small sample size. This work still needs more samples and even replicates (both Cross-platform replicates and intra-platform replicates) to do further analysis, and provide confident evidence.
2. The samples for sequencing were extracted on different points of time from the individual, that we wonder if the differences between mutation sets of seven sequencing platforms were caused by different sampling time and the bias of sampling process.
3. This manuscript needs to show more detail about the sequencing process, such as the number of the flow cell and sequencing cycle, the run time of the sequencing process, the amount of DNA each sequencing platform needs.
4. In order to compare, the sequencing data of seven sequencing platforms need to have the same genome coverage.
5. The results of the manuscript let me worry about the quality of the sequencing data generated from Hiseq2000 and Hiseq4000. More samples or replicates were needed to prove these results that the author found were normal.
6. According to the official information, MGI platforms have low duplicate rate than any sequencing platform which needs PCR. But this work showed MGISEQ T7 had highest duplicate rate, I suggest the authors prove their finding by using other samples or individuals.
7. The methods for identifying the platform-specific covered region are unreasonable as different sequencing platforms had different coverage.
8. The Comparison of variants detected among seven platforms needs further analysis. Authors need a standard SNP and indel list of the Korean reference genome, which is verified by Sanger sequencing or other methods, to replace the dbSNP and SNP genotype chip as a compare object. What the relationship of FP, FN and the sequencing errors?
9. The introduction of this manuscript is too simple.

Minor revisions:

1. The coverages of BGISEQ-500 and HiseqX10 were not mentioned in the first section.
2. Using the ratio of singletons may help you to bring out your findings more clearly.

Level of Interest

Please indicate how interesting you found the manuscript: Choose an item.

Quality of Written English

Please indicate the quality of language in the manuscript: Choose an item.

Declaration of Competing Interests

Please complete a declaration of competing interests, considering the following questions:

- Have you in the past five years received reimbursements, fees, funding, or salary from an organisation that may in any way gain or lose financially from the publication of this manuscript, either now or in the future?
- Do you hold any stocks or shares in an organisation that may in any way gain or lose financially from the publication of this manuscript, either now or in the future?
- Do you hold or are you currently applying for any patents relating to the content of the manuscript?
- Have you received reimbursements, fees, funding, or salary from an organization that holds or has applied for patents relating to the content of the manuscript?
- Do you have any other financial competing interests?
- Do you have any non-financial competing interests in relation to this paper?

If you can answer no to all of the above, write 'I declare that I have no competing interests' below. If your reply is yes to any, please give details below.

I declare that I have no competing interests

I agree to the open peer review policy of the journal. I understand that my name will be included on my report to the authors and, if the manuscript is accepted for publication, my named report including any attachments I upload will be posted on the website along with the authors' responses. I agree for my report to be made available under an Open Access Creative Commons CC-BY license (http://creativecommons.org/licenses/by/4.0/). I understand that any comments which I do not wish to be included in my named report can be included as confidential comments to the editors, which will not be published.

Choose an item.

To further support our reviewers, we have joined with Publons, where you can gain additional credit to further highlight your hard work (see: https://publons.com/journal/530/gigascience). On publication of this paper, your review will be automatically added to Publons, you can then choose whether or not to claim your Publons credit. I understand this statement.

Yes Choose an item.
