# Supplementary material for: Comparative analysis of 7 short-read sequencing platforms using the Korean Reference Genome: MGI and Illumina sequencing benchmark for whole-genome sequencing
Source: Gigascience. 2021 Mar 12;10(3):giab014. doi: 10.1093/gigascience/giab014 (PMC7953489; doi:10.1093/gigascience/giab014)
Supplement: giab014_Reviewer_2_Report_Original_Submission — Xuming Zhou -- 5/29/2020 Reviewed [file giab014_reviewer_2_report_original_submission.pdf]

Reviewer Report

Title: Comparative analysis of seven short-read sequencing platforms using the Korean Reference Genome: MGI and Illumina sequencing benchmark for whole-genome sequencing

Version: Original Submission **Date:** 5/29/2020

Reviewer name: Xuming Zhou

Reviewer Comments to Author:

The submitted study has characterized sequencing quality, uniformity of coverage, %GC coverage, and variant accuracy of seven sequencing platforms. They found that MGI platforms showed a higher concordance rate of SNP genotyping than HiSeq series. The study is of interest to genomics and sequencing technologies areas. Two concerns must be addressed prior to acceptance.
1)The author defined low-quality reads as those that had more than 30% of bases with a sequencing quality score lower than 20. I am wondering whether the results is stable once the definition changed ?
2)It looks the author ignored a highest duplicate ratio was found in MGISEQ-T7. More discussion and analysis should be preformed to make this clear. The author claimed that duplicates and adapter contamination may be more affected by the process of sample preparation than by the sequencing instrument. However, again, no evidence was provided.

Level of Interest

Please indicate how interesting you found the manuscript: Choose an item.

Quality of Written English

Please indicate the quality of language in the manuscript: Choose an item.

Declaration of Competing Interests

Please complete a declaration of competing interests, considering the following questions:

- Have you in the past five years received reimbursements, fees, funding, or salary from an organisation that may in any way gain or lose financially from the publication of this manuscript, either now or in the future?
- Do you hold any stocks or shares in an organisation that may in any way gain or lose financially from the publication of this manuscript, either now or in the future?
- Do you hold or are you currently applying for any patents relating to the content of the manuscript?
- Have you received reimbursements, fees, funding, or salary from an organization that holds or has applied for patents relating to the content of the manuscript?
- Do you have any other financial competing interests?

- Do you have any non-financial competing interests in relation to this paper?

If you can answer no to all of the above, write 'I declare that I have no competing interests' below. If your reply is yes to any, please give details below.

no

I agree to the open peer review policy of the journal. I understand that my name will be included on my report to the authors and, if the manuscript is accepted for publication, my named report including any attachments I upload will be posted on the website along with the authors' responses. I agree for my report to be made available under an Open Access Creative Commons CC-BY license (http://creativecommons.org/licenses/by/4.0/). I understand that any comments which I do not wish to be included in my named report can be included as confidential comments to the editors, which will not be published.

Choose an item.

To further support our reviewers, we have joined with Publons, where you can gain additional credit to further highlight your hard work (see: https://publons.com/journal/530/gigascience). On publication of this paper, your review will be automatically added to Publons, you can then choose whether or not to claim your Publons credit. I understand this statement.

Yes Choose an item.
